# Robust sparse smooth principal component analysis for face reconstruction and recognition

**DOI:** 10.1371/journal.pone.0323281

**Published:** 2025-05-27

**Authors:** Jing Wang, Xiao Xie, Li Zhang, Jian Li, Hao Cai, Yan Feng

**Affiliations:** 1 School of Computer and Information Technology, Xinyang Normal University, Xinyang, Henan, China; 2 Henan Key Laboratory of Analysis and Applications of Education Big Data, Xinyang Normal University, Xinyang, Henan, China; 3 School of Early-Childhood Education, Nanjing Xiaozhuang University, Nanjing, Jiangsu, China; University of South Australia, AUSTRALIA

## Abstract

Existing Robust Sparse Principal Component Analysis (RSPCA) does not incorporate the two-dimensional spatial structure information of images. To address this issue, we introduce a smooth constraint that characterizes the spatial structure information of images into conventional RSPCA, generating a novel algorithm called Robust Sparse Smooth Principal Component Analysis (RSSPCA). The proposed RSSPCA achieves three key objectives simultaneously: robustness through L1-norm optimization, sparsity for feature selection, and smoothness for preserving spatial relationships. Within the Minorization-Maximization (MM) framework, an iterative process is designed to solve the RSSPCA optimization problem, ensuring that a locally optimal solution is achieved. To evaluate the face reconstruction and recognition performance of the proposed algorithm, we conducted comprehensive experiments on six benchmark face databases. Experimental results demonstrate that incorporating robustness and smoothness improves reconstruction performance, while incorporating sparsity and smoothness improves classification performance. Consequently, the proposed RSSPCA algorithm generally outperforms existing algorithms in face reconstruction and recognition. Additionally, visualization of the generalized eigenfaces provides intuitive insights into how sparse and smooth constraints influence the feature extraction process. The data and source code from this study have been made publicly available on the GitHub repository: https://github.com/yuzhounh/RSSPCA.

## 1. Introduction

Principal Component Analysis (PCA) [1,2] has been widely applied in dimensionality reduction, signal reconstruction, and pattern classification [3–5]. However, traditional PCA adopts L2-norm in the objective function, which is easily affected by noise. Applying L1-norm to the objective function of PCA generates the PCA with L1-norm (PCA-L1) [6], which is robust and can effectively reduce the influence of data noise.

Sparsity [7,8] is another important property. Sparse modelling can automatically find relevant features from training data while ignoring irrelevant features. It not only improves the generalization ability of an algorithm but also increases the interpretability of the results. Therefore, sparse modelling has been widely applied in signal processing, machine learning, pattern recognition, and many other fields [9,10]. Traditional PCA cannot extract sparse principal components. To address this issue, L1-norm is applied to the constraint function of traditional PCA, generating the Sparse PCA (SPCA) [11]. Due to the sparsity-promoting property of L1-norm, the principal components extracted by SPCA are sparse.

Inspired by PCA-L1 and SPCA, Robust SPCA (RSPCA) [12] applies L1-norm to both the objective and constraint functions of traditional PCA for simultaneously robust and sparse modelling. However, when processing facial images using PCA and its variants, these images must be reshaped into vectors before further processing, which inevitably leads to the loss of inherent two-dimensional spatial structure information. While Two-dimensional PCA (2DPCA) [13] and its improved variants [14–16] attempt to address this limitation by expressing face images as matrices, they still do not fully capture and utilize the rich spatial structure information present in image data.

Various approaches have been developed to effectively preserve and utilize spatial structure information in image processing tasks. These include texture analysis methods that capture local patterns and regularities [17], graph-based techniques that model relationships between image regions [18,19], geometric approaches that analyze shapes and spatial configurations [20], deep learning architectures specifically designed to maintain spatial correlations [21], etc. These approaches leverage the inherent continuity and gradual variations present in natural images to preserve essential spatial relationships. Among these methods, smoothness-based approaches [22] have demonstrated particular effectiveness in handling data with rich spatial and temporal structural characteristics.

The smooth constraint [23] characterizes the interactions among adjacent features and has achieved remarkable success in brain decoding applications, where preserving spatial and temporal relationships is crucial. It was initially introduced in Electroencephalogram (EEG) decoding [23], where it proved essential for capturing the continuity of brain activity patterns. Subsequently, the combination of sparseness and smoothness was fully investigated by [24]. Its effectiveness led to widespread adoption in various neuroimaging applications, including Magnetoencephalography (MEG) [25], functional Magnetic Resonance Imaging (fMRI) [26–28], and Electrocorticographic (ECoG) [29] decoding. Recently, smoothness was also applied in many other fields, including functional connectivity-based brain region parcellation [30], hyperspectral image classification [31], reconstruction of compressively sensed multichannel EEG signals [32], foreground estimation in neuronal images [33], etc. The success in these applications stems from the constraint’s ability to model and preserve the intrinsic spatial and temporal relationships within the data, which is particularly crucial when dealing with complex, structured information.

The implementation of smoothness is typically achieved through a graph Laplacian matrix [34], which effectively represents the spatial and temporal structure information of data. This approach is rooted in spectral graph theory [35,36], where Laplacian eigenmaps capture the intrinsic geometry of high-dimensional data [37,38]. While traditional dimensionality reduction techniques like Laplacian eigenmaps [39,40] have been widely used in machine learning and pattern recognition, they primarily focus on general data representation rather than explicitly preserving the spatial and temporal relationships between adjacent features.

Building upon these insights and the success of smooth constraints in brain decoding [23–33], we propose to incorporate this constraint into conventional RSPCA, resulting in a novel approach termed Robust Sparse Smooth PCA (RSSPCA). This integration addresses a major limitation of RSPCA, i.e., its inability to account for spatial structure information in images. The proposed RSSPCA achieves three key objectives simultaneously: robustness through L1-norm optimization, sparsity for feature selection, and smoothness for preserving spatial relationships. These combined properties make RSSPCA particularly well-suited for face image processing tasks, where preserving spatial structure is crucial for accurate reconstruction and recognition.

The synergy between sparsity and smoothness in RSSPCA is theoretically justified by their complementary nature [24,41]. While sparsity helps identify the most relevant features and reduces noise, smoothness ensures that the spatial coherence of these features is maintained, leading to more naturalistic and interpretable results. This combination has proven particularly effective in applications where both feature selection and structural preservation are important, such as in brain decoding and image processing tasks.

To validate the effectiveness of RSSPCA, we conducted comprehensive experiments on six benchmark face databases, comparing its performance with four competing algorithms in terms of face reconstruction and recognition accuracy. The results demonstrate that RSSPCA significantly outperforms existing methods, confirming the advantages of incorporating smooth constraints into robust sparse PCA frameworks.

The remainder of this paper is organized as follows. Section 2 reviews traditional PCA and its robust and sparse variants. Section 3 presents our proposed RSSPCA methodology, including the problem formulation, related techniques, and iterative solutions. Section 4 demonstrates the effectiveness of our approach through comprehensive experiments on face reconstruction and recognition. Section 5 discusses the limitations of our approach and suggests directions for future research. Finally, Section 6 concludes the paper.

## 2. Related works

In this paper, lowercase letters represent scalars, boldface lowercase letters represent column vectors, boldface uppercase letters represent matrices; $\|\cdot {\;\|}_{1}$, $\|\cdot {\;\|}_{2}$, and $\|\cdot {\;\|}_{F}$ represent L1-norm, L2-norm, and Frobenius norm, respectively; sign(·) represents the sign function; diag(a) represents a square diagonal matrix with the elements of vector a on the main diagonal.

This section reviews the traditional PCA and its robust and sparse variants. For the variants of PCA, we emphasize on finding the first projection vector. After that, multiple projection vectors can be extracted by implementing a deflation scheme.

### 2.1 PCA

Let $X=\left[x_{1},x_{2},\ldots ,x_{n}\right]\in {\mathbb{R}}^{d*n}$ be n training images where each row is a feature and each column is an image. The images are assumed to be mean-centered, i.e., $\frac{1}{n}\sum_{i=1}^{n}x_{i}=0$. PCA [1,2] finds the first principal component $w\in {\mathbb{R}}^{d*1}$ by solving the following optimization problem:


$$\begin{array}{c} \begin{array}{c} \max_{w}{\left\|X^{T}w\right\|}_{2}^{2},\;s.t.\;\;{\left\|w\right\|}_{2}^{2}=1. \end{array} \end{array}$$ (1)

The projection vector w can be obtained by conducting eigen decomposition of the image covariance matrix and preserving the eigenvector with the largest eigenvalue.

### 2.2 PCA-L1

PCA with L1-norm (PCA-L1) [6] is formulated by replacing the L2-norm in the objective function of PCA with L1-norm. That is, PCA-L1 finds the first projection vector by solving the following optimization problem:


$$\begin{array}{c} \begin{array}{c} \max_{w}{\left\|X^{T}w\right\|}_{1},\;s.t.\;\;{\left\|w\right\|}_{2}^{2}=1. \end{array} \end{array}$$ (2)

The projection vector w can be computed by an iterative procedure. Let k be the iteration number, $w^{\left(k\right)}$ be the projection vector at the kth step, then w is updated by:


$$\begin{array}{c} v^{\left(k\right)}=Xsign\left(X^{T}w^{\left(k\right)}\right), \end{array}$$ (3)

$$\begin{array}{c} w^{\left(k+1\right)}=\frac{v^{\left(k\right)}}{{\left\|v^{\left(k\right)}\right\|}_{2}}. \end{array}$$ (4)

By incorporating L1-norm into the objective function of PCA, the resulting PCA-L1 algorithm demonstrates enhanced robustness against the impact of data noise.

### 2.3 RSPCA

Robust Sparse PCA (RSPCA) [12] is formulated by incorporating the L1-norm into both the objective and constraint functions of PCA. That is, RSPCA finds the first projection vector by solving the following optimization problem:


$$\begin{array}{c} \begin{array}{c} \max_{w}{\left\|X^{T}w\right\|}_{1},\;s.t.\;\;{\left\|w\right\|}_{2}^{2}=1,\;{\left\|w\right\|}_{1}\leq c \end{array}, \end{array}$$ (5)

where c is a positive constant. RSPCA has two different iterative solutions. The first solution [12] only relaxes the objective function and then solves the relaxed optimization problem by soft-thresholding [42]. That is, the projection vector w is updated by:


$$\begin{array}{c} v^{\left(k\right)}=Xsign\left(X^{T}w^{\left(k\right)}\right), \end{array}$$ (6)

$$\begin{array}{c} u^{\left(k\right)}=soft\left(v^{\left(k\right)},\lambda \right)=sign\left(v^{\left(k\right)}\right){\left(\left|v^{\left(k\right)}\right|-\lambda \right)}_{+}, \end{array}$$ (7)

$$\begin{array}{c} w^{\left(k+1\right)}=\frac{u^{\left(k\right)}}{{\left\|u^{\left(k\right)}\right\|}_{2}}, \end{array}$$ (8)

where soft(v,λ) is the soft-thresholding operator, $\left(x{)}_{+}=max(x,\;0\right)$, λ is the Lagrange multiplier. The sign(·) and $(\cdot {)}_{+}$ functions can be applied on a vector in an elementwise manner. Inspired by [14,15], RSPCA can also be solved by simultaneously relaxing the objective and constraint functions. That is, the projection vector w is updated by:


$$\begin{array}{c} v^{\left(k\right)}=Xsign\left(X^{T}w^{\left(k\right)}\right), \end{array}$$ (9)

$$\begin{array}{c} u_{i}^{\left(k\right)}=v_{i}^{\left(k\right)}\frac{\left|w_{i}^{\left(k\right)}\right|}{\lambda +\left|w_{i}^{\left(k\right)}\right|},\;i=1,2,\ldots ,d, \end{array}$$ (10)

$$\begin{array}{c} w^{\left(k+1\right)}=\frac{u^{\left(k\right)}}{{\left\|u^{\left(k\right)}\right\|}_{2}}. \end{array}$$ (11)

where $w_{i}^{\left(k\right)}$, $v_{i}^{\left(k\right)}$, and $u_{i}^{\left(k\right)}$ are the ith elements of $w^{\left(k\right)}$, $v^{\left(k\right)}$, and $u^{\left(k\right)}$, respectively. The parameter λ is a nonnegative scalar that adjusts the relative weight between the sparse constraint and the L2-norm constraint. When λ is set to zero, the L1-norm constraint becomes invalid and RSPCA reduces to PCA-L1. When λ increases, the sparsity of the projection vector w increases. When λ is set to positive infinity, the L2-norm constraint becomes invalid. As a result, the projection vector w becomes a vector with only one nonzero element that equals one [15,43]. This is the reason why the L2-norm constraint is reserved in RSPCA [12] and its 2D counterpart, i.e., 2DPCA-L1 with Sparsity (2DPCAL1-S) [14]. Due to the L1-norm in the objective and constraint functions, RSPCA achieves robustness and sparseness simultaneously.

After obtaining the first r projection vectors $W=[w_{1},\;w_{2},\;\ldots ,w_{r}]\in {\mathbb{R}}^{d*r}$ for PCA, PCA-L1, or RSPCA, 1≤r<d, the (r+1)th projection vector $w_{r+1}$ is calculated likewise on the deflated sample [44]


$$\begin{array}{c} X^{deflated}=\left(I-WW^{T}\right)X. \end{array}$$ (12)

By iteratively implementing the deflation procedure, we can extract multiple projection vectors [6,12].

## 3. Proposed methodology

This section presents our proposed Robust Structured Sparse PCA (RSSPCA) algorithm. We first formulate the optimization problem and define the objective function. Then, we introduce the Laplacian matrix and its associated smoothness penalty term to capture spatial relationships. Next, we elaborate on the Minorization-Maximization (MM) framework that underpins our solution approach, followed by two essential inequalities that facilitate our theoretical derivation. Finally, based on the MM framework and the introduced inequalities, we develop a complete iterative solution to the RSSPCA optimization problem.

### 3.1 Problem formulation

Based on the robust and sparse PCA algorithms presented above, we propose RSSPCA by incorporating a smooth constraint. That is, RSSPCA finds its first projection vector by solving the following optimization problem:


$$\begin{array}{c} \begin{array}{c} \max_{w}{\left\|X^{T}w\right\|}_{1},\;s.t.\;\;{\left\|w\right\|}_{2}^{2}=1,\;{\left\|w\right\|}_{1}\leq c_{1},\;w^{T}Lw\leq c_{2}, \end{array} \end{array}$$ (13)

where $c_{1}$ and $c_{2}$ are positive constants, L is a Laplacian matrix representing the two-dimensional spatial structure information of images.

The optimization problem of RSSPCA has four parts. The first part ${\left\|X^{T}w\right\|}_{1}$ is the objective function. The L1-norm in the objective function makes RSSPCA robust against noise. The second part ${\left\|w\right\|}_{2}^{2}=1$ is the L2-norm constraint. It is reserved for the same reason as in RSPCA [12] and 2DPCAL1-S [14]. The third part ${\left\|w\right\|}_{1}\leq c_{1}$ is the sparse constraint, which makes the projection vector w sparse. The fourth part $w^{T}Lw\leq c_{2}$ is the smooth constraint, which makes the projection vector w spatially smooth. Therefore, RSSPCA is modelled to be robust, sparse, and smooth at the same time. It is anticipated to offer superior performance in face reconstruction and recognition tasks when compared to PCA and its variants.

### 3.2 Laplacian matrix

**Fig 1:**
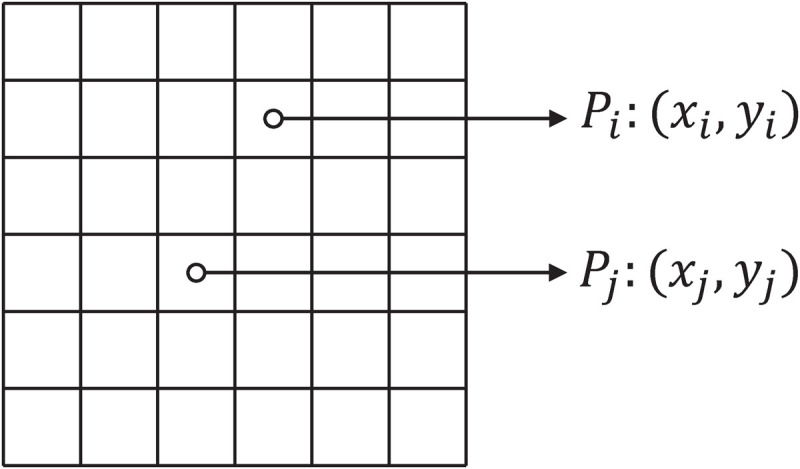
An illustration of the coordinates of two pixels in an image.

The Laplacian Eigenmaps approach [35,36] was initially proposed to capture the intrinsic geometric structure of low-dimensional manifolds. However, in the optimization problem of RSSPCA, we employ the Laplacian matrix to represent the two-dimensional spatial structure information inherent in images and subsequently construct the smoothness constraint. The definition of the Laplacian matrix is elaborated as follows. Suppose there are two pixels $P_{i}$ and $P_{j}$ on an image. Their coordinates on the two-dimensional plane are represented as $\left(x_{i},y_{i}\right)$ and $\left(x_{j},y_{j}\right)$, respectively, as illustrated in Fig 1.

The Euclidean distance between the two pixels is


$$\begin{array}{c} \begin{array}{c} d_{ij}={\left\|\left(x_{i},y_{i}\right)-\left(x_{j},y_{j}\right)\right\|}_{2}. \end{array} \end{array}$$ (14)

Define an adjacency matrix $A\in {\mathbb{R}}^{d*d}$ whose elements are


$$\begin{array}{c} \begin{array}{c} A_{ij}=\left\{ \begin{array}{cc} 1, & d_{ij}=1,\\ 0, & others, \end{array}\right.\; \end{array} \end{array}$$ (15)

i,j=1,2,…,d. That is, the corresponding element in the adjacency matrix A is one if and only if two pixels are adjacent on the two-dimensional plane, otherwise it is 0. The degree matrix D is defined based on the adjacency matrix as:


$$\begin{array}{c} \begin{array}{c} \begin{array}{c} D=diag\left(1_{d}^{T}A\right) \end{array} \end{array}, \end{array}$$ (16)

where $1_{d}\in {\mathbb{R}}^{d\times 1}$ represents a d-dimensinal column vector with all elements equal to one. The degree matrix D is a diagonal matrix. Each element on the main diagonal of D equals the number of pixels adjacent to the current pixel. Then the Laplacian matrix L is calculated based on the adjacency matrix and the degree matrix as


$$\begin{array}{c} \begin{array}{c} \begin{array}{c} L=D-A \end{array} \end{array}. \end{array}$$ (17)

**Fig 2:**
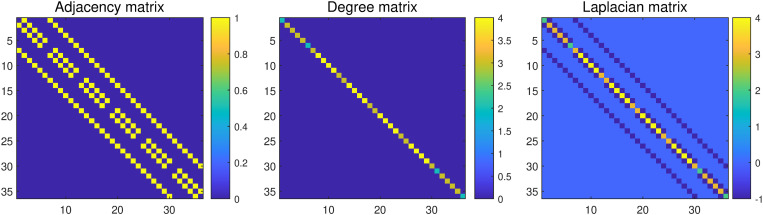
An illustration of the adjacency matrix, degree matrix, and Laplacian matrix.

Fig 2 shows examples of the adjacency matrix, degree matrix, and Laplacian matrix constructed by reshaping a 6×6 image into a vector.

According to the definition of Laplacian matrix, we have


$$\begin{array}{c} \begin{array}{c} w^{T}Lw=w^{T}\left(D-A\right)w=w^{T}\left(diag\left(1_{d}^{T}A\right)-A\right)w=\frac{1}{2}\sum_{i,j=1}^{d}{\left(w_{i}-w_{j}\right)}^{2}A_{ij} \end{array}. \end{array}$$ (18)

For two adjacent pixels i and j, $A_{ij}$ equals one, otherwise $A_{ij}$ equals zero. By incorporating this constraint, the difference between $w_{i}$ and $w_{j}$ is punished. That is, the weights corresponding to two adjacent pixels will be close. Therefore, this constraint achieves a smoothing effect on the projection vector.

### 3.3 Minorization-Maximization framework

The optimization problem of RSSPCA is challenging to solve due to the presence of L1-norm. This paper adopts the Minorization-Maximization (MM) framework [45] to address this issue. Suppose f(w) is the objective function to be maximized. Within the MM framework, if there exists a surrogate function $g\left(w|w^{\left(k\right)}\right)$ that satisfies the following two key conditions:


$$\begin{array}{c} \begin{array}{c} f\left(w^{\left(k\right)}\right)=g\left(w^{\left(k\right)}|w^{\left(k\right)}\right) \end{array}, \end{array}$$ (19)

$$\begin{array}{c} \begin{array}{c} f\left(w\right)\geq g\left(w|w^{\left(k\right)}\right),\;\forall w \end{array}, \end{array}$$ (20)

the original objective function f(w) can be optimized by iteratively maximizing the surrogate function $g\left(w|w^{\left(k\right)}\right)$ as follows:


$$\begin{array}{c} w^{\left(k+1\right)}=\mathrm{\backslash arg}\max_{w}g\left(w|w^{\left(k\right)}\right). \end{array}$$ (21)

Then we have


$$\begin{array}{l} f\left(w^{\left(k+1\right)}\right)=f\left(w^{\left(k+1\right)}\right)-g\left(w^{\left(k+1\right)}|w^{\left(k\right)}\right)+g\left(w^{\left(k+1\right)}|w^{\left(k\right)}\right)\;\\ \;\geq f\left(w^{\left(k\right)}\right)-g\left(w^{\left(k\right)}|w^{\left(k\right)}\right)+g\left(w^{\left(k+1\right)}|w^{\left(k\right)}\right)\;\\ \;\geq f\left(w^{\left(k\right)}\right)-g\left(w^{\left(k\right)}|w^{\left(k\right)}\right)+g\left(w^{\left(k\right)}|w^{\left(k\right)}\right)\\ \;=f\left(w^{\left(k\right)}\right)\;. \end{array}$$ (22)

The first inequality holds because $f\left(w\right)-g\left(w|w^{\left(k\right)}\right)$ reaches the minimum value at ${w=w}^{\left(k\right)}$ according to the two key conditions. The second inequality holds because $g\left(w|w^{\left(k\right)}\right)$ reaches the maximum value at ${w=w}^{\left(k+1\right)}$ according to the update rule. Therefore, the objective function f(w) monotonically increases during the iterative process and will converge to a local optimum. By finding a surrogate function that is easy to handle, we can solve the optimization problem of RSSPCA within the MM framework.

### 3.4 Inequality

The surrogate function is typically formulated by introducing inequalities. Below are two inequalities that will be used to solve RSSPCA. Let $X\in {\mathbb{R}}^{d*n}$, $w\in {\mathbb{R}}^{d*1}$, $w^{\left(k\right)}\in {\mathbb{R}}^{d*1}$, the following two inequalities


$$\begin{array}{c} \begin{array}{c} {\left\|X^{T}w\right\|}_{1}\geq {sign\left(X^{T}w^{\left(k\right)}\right)}^{T}X^{T}w, \end{array} \end{array}$$ (23)

$$\begin{array}{c} \begin{array}{c} {\left\|w\right\|}_{1}\leq \frac{1}{2}w^{T}diag\left({\left|w^{\left(k\right)}\right|}^{-1}\right)w+\frac{1}{2}{\left\|w^{\left(k\right)}\right\|}_{1}, \end{array} \end{array}$$ (24)

hold and the inequalities become equalities when $w=w^{\left(k\right)}$. The proofs for the two inequalities can be found in Wang [15]. Note that Equation (24) requires that $w^{\left(k\right)}$ must not contain zero elements.

### 3.5 Solution

Based on the MM framework and the introduced inequalities, we can develop an iterative solution to the RSSPCA optimization problem. Maximizing the optimization problem of RSSPCA equals maximizing the Lagrangian as follows:


$$\begin{array}{c} \max_{w}{\left\|X^{T}w\right\|}_{1}-\lambda_{1}\left({\left\|w\right\|}_{2}^{2}-1\right)-\lambda_{2}\left({\left\|w\right\|}_{1}-c_{1}\right)-\lambda_{3}\left(w^{T}Lw-c_{2}\right)\begin{array}{c} , \end{array} \end{array}$$ (25)

where $\lambda_{1}$, $\lambda_{2}$, and $\lambda_{3}$ are three Lagrangian multipliers satisfying that $\lambda_{1}>0$, $\lambda_{2}\geq 0$, and $\lambda_{3}\geq 0$. Denote the Lagrangian as f(w). According to Equations (23) and (24),


$$\begin{array}{l} {\left\|X^{T}w\right\|}_{1}-\lambda_{1}\left({\left\|w\right\|}_{2}^{2}-1\right)-\lambda_{2}\left({\left\|w\right\|}_{1}-c_{1}\right)-\lambda_{3}\left(w^{T}Lw-c_{2}\right)\\ \geq sign{\left(X^{T}w^{\left(k\right)}\right)}^{T}X^{T}w-\lambda_{1}\left({\left\|w\right\|}_{2}^{2}-1\right)\\ -\lambda_{2}\left(\frac{1}{2}w^{T}diag\left({\left|w^{\left(k\right)}\right|}^{-1}\right)w+\frac{1}{2}{\left\|w^{\left(k\right)}\right\|}_{1}-c_{1}\right)-\lambda_{3}\left(w^{T}Lw-c_{2}\right) \end{array}$$ (26)

holds and the inequality becomes equality when $w=w^{\left(k\right)}$. Denote the relaxed function as $g\left(w|w^{\left(k\right)}\right)$. We have $f\left(w^{\left(k\right)}\right)=g\left(w^{\left(k\right)}|w^{\left(k\right)}\right)$ and $\begin{array}{c} f\left(w\right)\geq g\left(w|w^{\left(k\right)}\right) \end{array}$ for all w, satisfying the two key conditions of the MM framework. Therefore, $g\left(w|w^{\left(k\right)}\right)$ is a feasible surrogate function of the Lagrangian f(w). Within the MM framework, maximizing f(w) can be turned into iteratively maximizing the surrogate function $g\left(w|w^{\left(k\right)}\right)$ as follows:


$$\begin{array}{l} w^{\left(k+1\right)}=\arg\max_{w}g\left(w|w^{\left(k\right)}\right)\\ \;=\arg\max_{w}-w^{T}\left(\lambda_{1}I+\frac{\lambda_{2}}{2}diag\left({\left|w^{\left(k\right)}\right|}^{-1}\right)+\lambda_{3}L\right)w\;\\ \;+{\left(Xsign\left(X^{T}w^{\left(k\right)}\right)\right)}^{T}w-\frac{\lambda_{2}}{2}{\left\|w^{\left(k\right)}\right\|}_{1}+\lambda_{1}+\lambda_{2}c_{1}+\lambda_{3}c_{2}. \end{array}$$ (27)

It is a quadratic optimization problem concerning to w. Its solution is


$$\begin{array}{c} w^{\left(k+1\right)}=\frac{1}{2}{\left(\lambda_{1}I+\frac{\lambda_{2}}{2}{U^{\left(k\right)}}^{-1}+\lambda_{3}L\right)}^{-1}v^{\left(k\right)}, \end{array}$$ (28)

where $U^{\left(k\right)}=diag\left(\left|w^{\left(k\right)}\right|\right)$, $v^{\left(k\right)}=Xsign\left(X^{T}w^{\left(k\right)}\right)$. Considering that ${\left\|w\right\|}_{2}^{2}=1$, we have


$$\begin{array}{c} w^{\left(k+1\right)}=\frac{{\left(\lambda_{1}I+\frac{\lambda_{2}}{2}{U^{\left(k\right)}}^{-1}+\lambda_{3}L\right)}^{-1}v^{\left(k\right)}}{{\left\|{\left(\lambda_{1}I+\frac{\lambda_{2}}{2}{U^{\left(k\right)}}^{-1}+\lambda_{3}L\right)}^{-1}v^{\left(k\right)}\right\|}_{2}}. \end{array}$$ (29)

Let $\eta_{1}=\frac{\lambda_{2}}{2\lambda_{1}}$ and $\eta_{2}=\frac{\lambda_{3}}{\lambda_{1}}$, then $\eta_{1}\geq 0$, $\eta_{2}\geq 0$. Equation (29) can be rewritten as:


$$\begin{array}{c} \begin{array}{c} w^{\left(k+1\right)}=\frac{{\left(I+\eta_{1}{U^{\left(k\right)}}^{-1}+\eta_{2}L\right)}^{-1}v^{\left(k\right)}}{{\left\|{\left(I+\eta_{1}{U^{\left(k\right)}}^{-1}+\eta_{2}L\right)}^{-1}v^{\left(k\right)}\right\|}_{2}} \end{array}. \end{array}$$ (30)

Therefore, it is only necessary to tune two parameters, i.e., $\eta_{1}$ and $\eta_{2}$, in RSSPCA. The parameter $\eta_{1}$ tunes the relative weight between the sparse constraint and the L2-norm constraint. The parameter $\eta_{2}$ tunes the relative weight between the smooth constraint and the L2-norm constraint. In summary, the two parameters adjust the relative weights of the three constraints in the optimization problem of RSSPCA.

Considering that $w^{\left(k\right)}$ might be a sparse vector, calculating ${U^{\left(k\right)}}^{-1}$ could encounter division by zero errors. To avoid this problem, we rewrite Equation (30) as follows:


$$\begin{array}{c} \begin{array}{c} w^{\left(k+1\right)}=\frac{{\left(U^{\left(k\right)}+\eta_{1}I+\eta_{2}U^{\left(k\right)}L\right)}^{-1}U^{\left(k\right)}v^{\left(k\right)}}{{\left\|{\left(U^{\left(k\right)}+\eta_{1}I+\eta_{2}U^{\left(k\right)}L\right)}^{-1}U^{\left(k\right)}v^{\left(k\right)}\right\|}_{2}} \end{array}. \end{array}$$ (31)

This update rule no longer requires that there are no zero elements in $w^{\left(k\right)}$. Equation (31) can be reformulated in the following two-step form:


$$\begin{array}{c} \begin{array}{c} u^{\left(k\right)}={\left(U^{\left(k\right)}+\eta_{1}I+\eta_{2}U^{\left(k\right)}L\right)}^{-1}U^{\left(k\right)}v^{\left(k\right)} \end{array}, \end{array}$$ (32)

$$\begin{array}{c} \begin{array}{c} w^{\left(k+1\right)}=\frac{u^{\left(k\right)}}{{\left\|u^{\left(k\right)}\right\|}_{2}} \end{array}. \end{array}$$ (33)

Update w iteratively until convergence, the result is the first projection vector of RSSPCA. Then, multiple projection vectors can be extracted likewise by iteratively implementing the deflation strategy [44]. The algorithm procedure of RSSPCA is listed in Algorithm 1.

**Algorithm 1. The algorithm procedure of RSSPCA.**

**Input:** training samples $X\in {\mathbb{R}}^{d*n}\$$, number of projection vectors r$, parameters $\eta_{1}\$$ and $\eta_{2}\$$.

**Output:** projection vector $W\in {\mathbb{R}}^{d*r}\$$.

**for** i=1:r$

   initialize k=0$, δ=1$.

   Initialize $w^{\left(0\right)}\$$ by the i$th principal component.

   $f^{\left(0\right)}={\left\|X^{T}w^{\left(0\right)}\right\|}_{1}\$$.

   **while** $\delta >10^{-4}\$$ **and** k<100$

             $U^{\left(k\right)}=diag\left(\left|w^{\left(k\right)}\right|\right)\$$.

             $v^{\left(k\right)}=Xsign\left(X^{T}w^{\left(k\right)}\right)\$$.

             $u^{\left(k\right)}={\left(U^{\left(k\right)}+\eta_{1}I+\eta_{2}U^{\left(k\right)}L\right)}^{-1}U^{\left(k\right)}v^{\left(k\right)}\$$.

             $w^{\left(k+1\right)}=\frac{u^{\left(k\right)}}{{\left\|u^{\left(k\right)}\right\|}_{2}}\$$.

             $f^{\left(k+1\right)}={\left\|X^{T}w^{\left(k+1\right)}\right\|}_{1}\$$.

             $\delta =\frac{\left|f^{\left(k+1\right)}-f^{\left(k\right)}\right|}{f^{\left(k\right)}}\$$.

             k⟵k+1$.

   **end while**

   $w_{i}=w^{\left(k+1\right)}\$$.

   $W=\left[w_{1},w_{2},\ldots ,w_{i}\right]\in {\mathbb{R}}^{d*i}\$$.

   $X⟵\left(I-WW^{T}\right)X\$$.

**end for**

From the optimization problem and the update rule of RSSPCA, it can be inferred that RSSPCA is a generalization of PCA-L1 and RSPCA. When $\eta_{1}=0$ and $\eta_{2}=0$, RSSPCA reduces to PCA-L1. When $\eta_{2}=0$, RSSPCA reduces to RSPCA. When $\eta_{1}=0$, RSSPCA reduces to Robust Smooth PCA (RSMPCA).

## 4. Experiments

**Fig 3:**
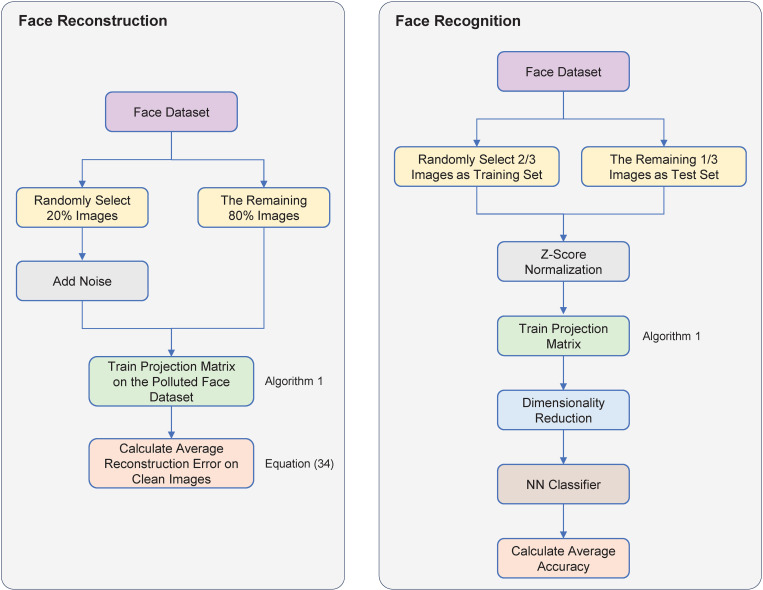
The flowchart of the experiments. The training of the projection matrix can be performed using the proposed RSSPCA method or any other competing algorithms.

This section presents comprehensive experimental evaluations of our proposed RSSPCA algorithm. We first assessed the reconstruction and recognition performance of RSSPCA against four competing algorithms in face reconstruction and recognition tasks. Subsequently, we visualized the projection vectors computed by different algorithms to provide intuitive insights into their underlying characteristics. Finally, we validated our approach on five additional publicly available benchmark face databases to demonstrate its robustness and generalizability across diverse datasets. The flowchart of the experiments is shown in Fig 3.

Our scripts were written in MATLAB and are publicly available at https://github.com/yuzhounh/RSSPCA. The experiments were conducted on four workstations, each equipped with dual 20-core 2.20 GHz Intel(R) Xeon(R) processors and 256 GB of memory. To minimize total computational time, we ran approximately 40 MATLAB sessions in parallel on each workstation. A parallel computing version of the code is available at https://github.com/yuzhounh/RSSPCA_parallel.

### 4.1 Face reconstruction

We first conducted a face reconstruction experiment to evaluate the reconstruction performance of RSSPCA and the four competing algorithms on the publicly available ORL Face Database [46,47], which was created and distributed by AT&T Laboratories Cambridge for research purposes. Four typical PCA-based algorithms, i.e., PCA [1,2], PCA-L1 [6], RSPCA [12], and RSMPCA, were compared with RSSPCA in the experiment.

The ORL face database contains 400 face images from 40 subjects, with 10 images per subject. The images were captured with different facial expressions, rotations, and slight scale variations. The original image size is 112 by 92. To reduce computational time, we further resized the images to 56 by 46. Among the 400 images in the ORL face database, 80 images were randomly selected and occluded with a rectangular salt-and-pepper noise whose size was not smaller than 10 by 10, located in a random position

Let W be the projection matrix trained on the polluted ORL face database, which includes 320 clean images and 80 occluded images. Let $Z_{1},Z_{2},\ldots ,Z_{m}$ be m clean images that are mean-centered, m=320. The average reconstruction error is defined as


$$\begin{array}{c} \begin{array}{c} \frac{1}{m}\sum_{i=1}^{m}{\left\|Z_{i}\left(I-WW^{T}\right)\right\|}_{F} \end{array}. \end{array}$$ (34)

It is used to evaluate the reconstruction performance of the five algorithms.

**Fig 4:**
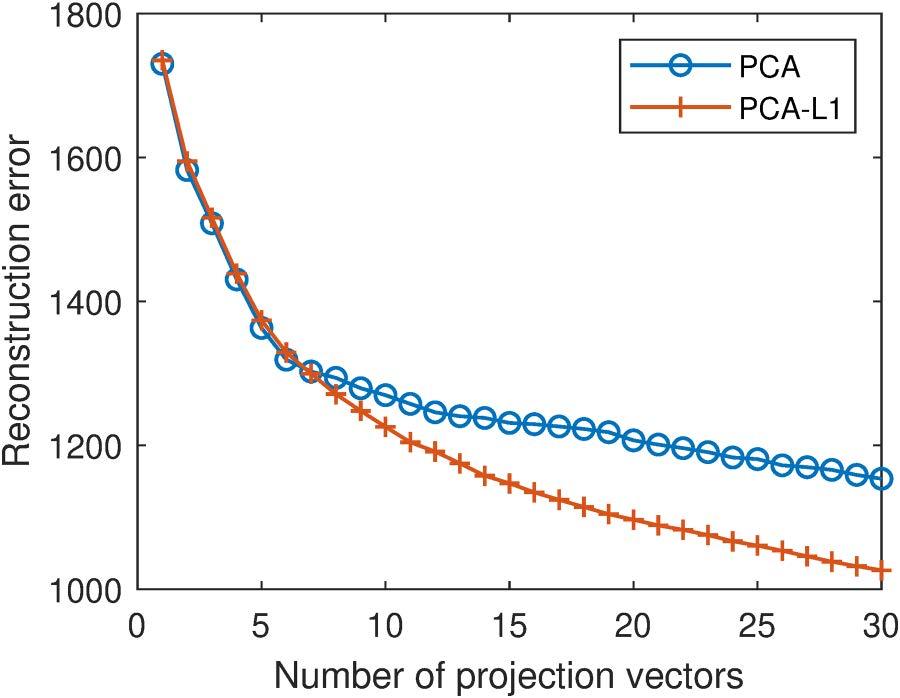
Reconstruction errors of PCA and PCA-L1 with different numbers of projection vectors.

Fig 4 shows the reconstruction errors of PCA and PCA-L1 with different numbers of projection vectors. In both cases, the reconstruction error monotonically decreases with increasing number of projection vectors. When the number of projection vectors is greater than 7, the reconstruction error of PCA-L1 is lower than that of PCA. By averaging the reconstruction errors with different projection vector numbers that are in the range of [1,30], we obtain the overall reconstruction errors of PCA and PCA-L1. They are 1272.33 and 1203.31, respectively. The results demonstrate that incorporating L1-norm into the objective function of PCA, i.e., incorporating robustness into PCA, can reduce reconstruction errors, consistent with the results in [6,14,15].

**Fig 5:**
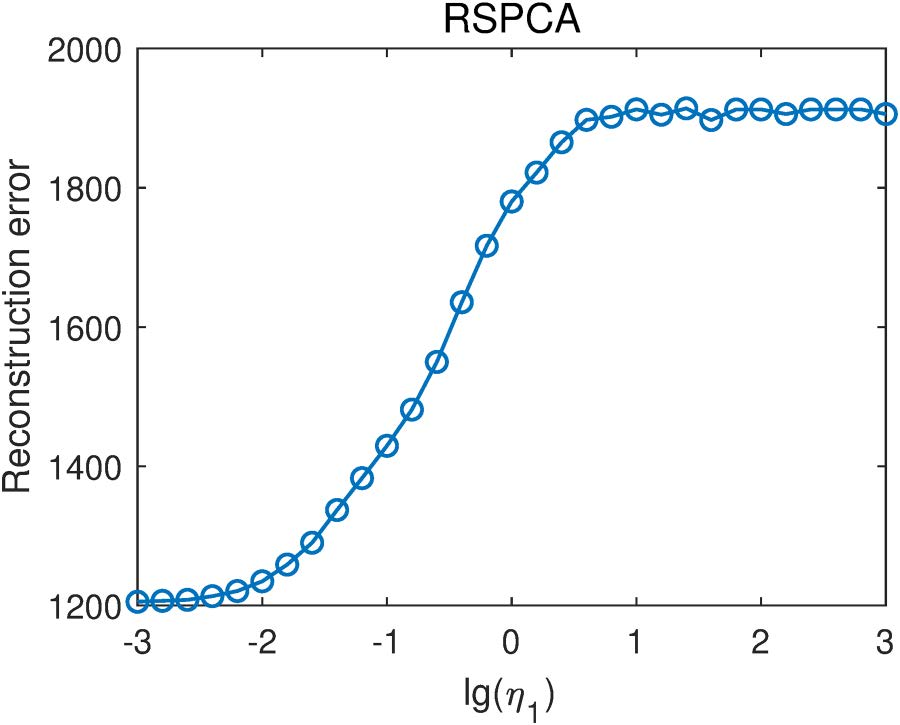
Reconstruction errors of RSPCA with different $\mathrm{lg}\left(\eta_{1}\right)$ values.

RSPCA is a special case of RSSPCA when $\eta_{2}=0$. Therefore, the projection vectors of RSPCA can be calculated by the update rule of RSSPCA. RSPCA only contains a parameter $\eta_{1}$ that tunes the relative weight between the sparse constraint and the L2-norm constraint. The parameters $\eta_{1}$ was selected from $\left\{10^{-3},10^{-2.8},10^{-2.6},\ldots ,10^{3}\right\}$. That is, $\mathrm{lg}\left(\eta_{1}\right)$ was selected from -3–3 with a step of 0.2. For each $\eta_{1}$ value, we averaged the reconstruction errors with different numbers of projection vectors to obtain the overall reconstruction errors, as shown in Fig 5. The overall reconstruction error generally increases with increasing $\mathrm{lg}\left(\eta_{1}\right)$ value. The lowest reconstruction error is 1205.41, obtained when $\mathrm{lg}\left(\eta_{1}\right)=-3.0$, very close to the reconstruction error of PCA-L1, which is 1203.31. From this result and the trend in Fig 5, we can infer that the lowest reconstruction error of RSPCA is achieved when $\eta_{1}=0$. In other words, RSPCA achieves the lowest reconstruction error when it reduces to PCA-L1. The results demonstrate that incorporating sparsity has little effect on reconstruction, consistent with the results in [14,15].

**Fig 6:**
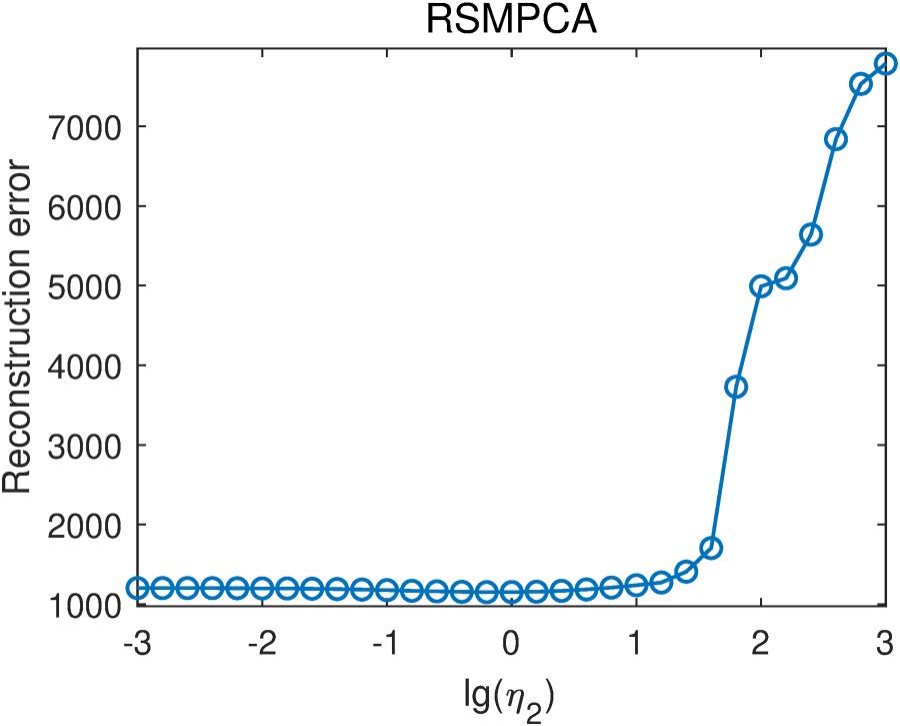
Reconstruction errors of RSMPCA with different $\mathrm{lg}\left(\eta_{2}\right)$ values.

RSMPCA is a special case of RSSPCA when $\eta_{1}=0$. Similarly, the projection vectors of RSMPCA can be calculated by the update rule of RSSPCA. RSMPCA contains a parameter $\eta_{2}$ that tunes the relative weight between the smooth constraint and the L2-norm constraint. The parameter $\eta_{2}$ was selected from $\left\{10^{-3},10^{-2.8},10^{-2.6},\ldots ,10^{3}\right\}$. That is, $\mathrm{lg}\left(\eta_{2}\right)$ was selected from -3–3 with a step of 0.2. For each $\eta_{2}$ value, we averaged the reconstruction errors with different projection vector numbers to obtain the overall reconstruction errors, as shown in Fig 6. When $\mathrm{lg}\left(\eta_{2}\right)\leq 1$, the reconstruction error was barely affected by $\mathrm{lg}\left(\eta_{2}\right)$. When $\mathrm{lg}\left(\eta_{2}\right)>1$, the reconstruction error significantly increases with increasing $\mathrm{lg}\left(\eta_{2}\right)$ value. The lowest reconstruction error is 1152.25, obtained when $\mathrm{lg}\left(\eta_{2}\right)=-0.2$. This result is lower than the reconstruction error of PCA-L1, indicating that incorporating smoothness improves reconstruction performance.

**Fig 7:**
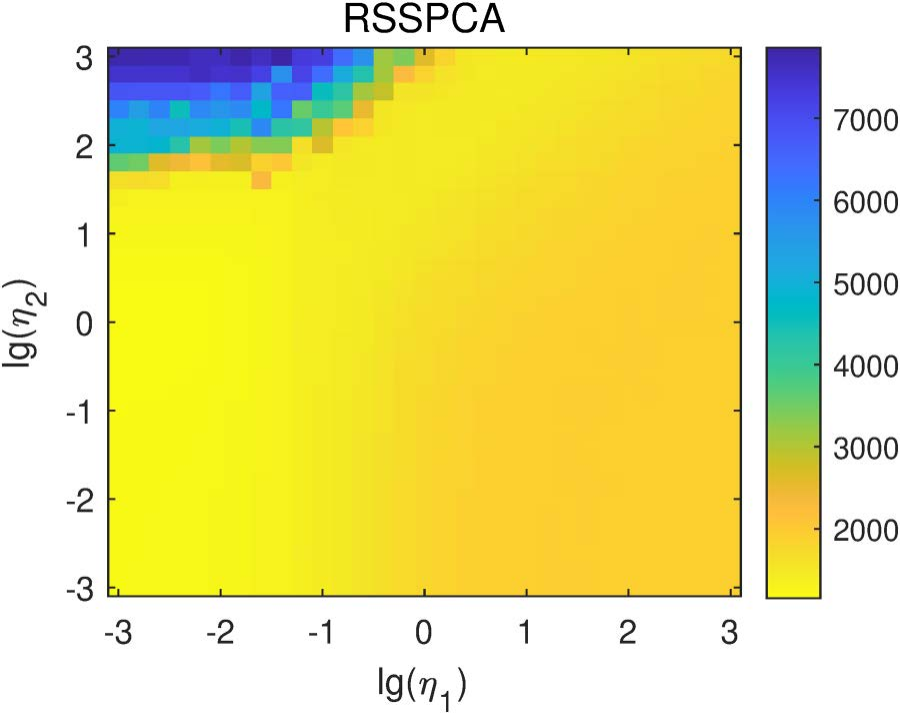
Reconstruction errors of RSSPCA with different $\mathrm{lg}\left(\eta_{1}\right)$ and $\mathrm{lg}\left(\eta_{2}\right)$ values.

RSSPCA contains two parameters $\eta_{1}$ and $\eta_{2}$, among which $\eta_{1}$ tunes the relative weight between the sparse constraint and the L2-norm constraint, and $\eta_{2}$ tunes the relative weight between the smooth constraint and the L2-norm constraint. For RSSPCA, both $\mathrm{lg}\left(\eta_{1}\right)$ and $\mathrm{lg}\left(\eta_{2}\right)$ were selected from -3–3 with a step of 0.2. The average reconstruction errors of RSSPCA under different parameters are shown in Fig 7. The lowest reconstruction error is 1153.35, obtained when $\mathrm{lg}\left(\eta_{1}\right)=-3.0$ and $\mathrm{lg}\left(\eta_{2}\right)=0$.

**Fig 8:**
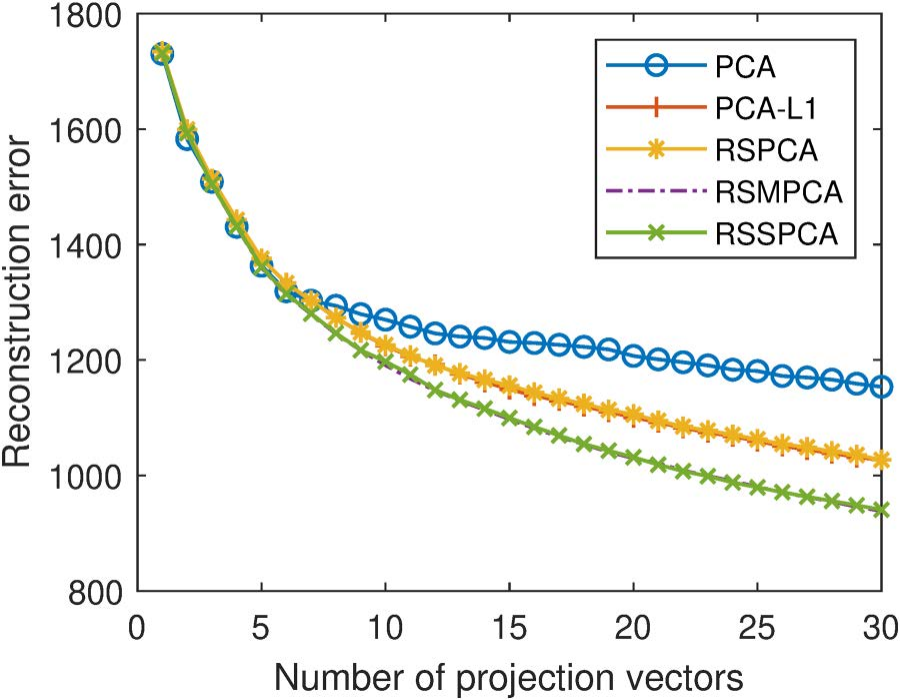
Reconstruction errors of the five algorithms with different numbers of projection vectors. The parameters are set to the optimal ones for each algorithm.

The lowest reconstruction errors and corresponding optimal parameters of the five algorithms are shown in Table 1. Fig 8 shows the reconstruction errors of the five algorithms with different number of projection vectors when the optimal parameters are selected.

**Table 1:** The lowest reconstruction errors and optimal parameters of the five algorithms.

Algorithm	Optimal parameters		Reconstruction error
	$\mathrm{lg}\left(\eta_{1}\right)$	$\mathrm{lg}\left(\eta_{2}\right)$	
PCA	/	/	1272.33
PCA-L1	/	/	1203.31
RSPCA	-3.0	/	1205.41
RSMPCA	/	-0.2	**1152.25**
RSSPCA	-3.0	0.0	1153.35

Based on the above results, it is evident that the reconstruction errors of PCA are the largest. PCA-L1 incorporates L1-norm into the objective function of PCA and achieves lower reconstruction errors than PCA, indicating that incorporating robustness is beneficial for reconstruction. RSPCA incorporates the sparse constraint into PCA-L1. The reconstruction errors of PCA-L1 and RSPCA are very close, indicating that incorporating sparsity has little effect on reconstruction. These results are consistent with the results in [6,14,15]. RSMPCA incorporates the smooth constraint into PCA-L1 and achieves lower reconstruction errors than PCA-L1, indicating that incorporating smoothness is beneficial for reconstruction. RSSPCA simultaneously incorporates the sparse constraint and the smooth constraint into PCA-L1. It is not surprising that RSSPCA achieves lower reconstruction errors than PCA-L1.

In addition, RSSPCA can be generated by incorporating the smooth constraint into RSPCA. With this adaptation, RSSPCA achieves lower reconstruction errors than RSPCA. It further demonstrates that incorporating smoothness is beneficial for reconstruction. From another perspective, RSSPCA can be generated by incorporating the sparse constraint into RSMPCA. But the reconstruction errors of RSSPCA and RSMPCA are very close. It further demonstrates that incorporating sparsity has little effect on reconstruction.

In summary, incorporating robustness and smoothness reduces reconstruction errors, while incorporating sparsity has little effect on reconstruction. These results demonstrate the superiority of RSSPCA over PCA, PCA-L1, and RSPCA in face reconstruction. When comparing RSSPCA and RSMPCA, they have similar reconstruction performance.

### 4.2 Face recognition

Next, we conducted a face recognition experiment to evaluate the classification performance of RSSPCA and the four competing algorithms. For each subject in the ORL face database, we randomly selected seven images for training and used the remaining three images for testing. The training images were normalized by z-score so that each feature was centered to have a mean of zero and scaled to have a standard deviation of one. The testing images were then normalized by applying the same parameters. After that, we applied the five algorithms to extract the first 30 projection vectors and applied these projection vectors to reduce the dimension of the normalized data. Finally, Nearest Neighbor (NN) classifier was applied to perform classification. The above procedure was repeated three times, and the average classification accuracy was calculated to evaluate the classification performance of the five algorithms.

**Fig 9:**
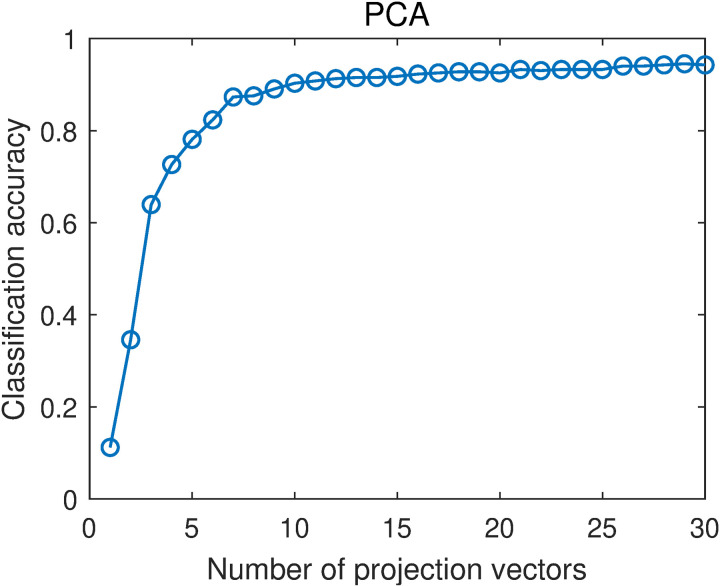
Average classification accuracies of PCA with different numbers of projection vectors.

**Fig 10:**
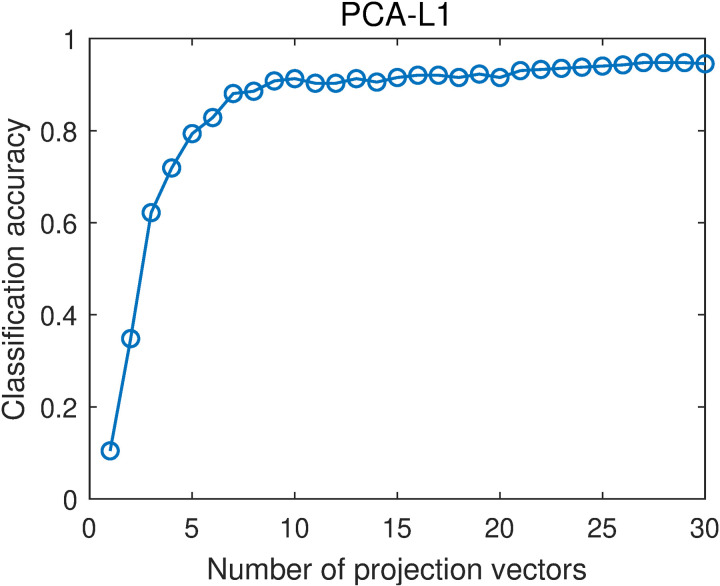
Average classification accuracies of PCA-L1 with different numbers of projection vectors.

Figs 9 and 10 show the average classification accuracies of PCA and PCA-L1 with different numbers of projection vectors. When the number of projection vectors increases, the classification accuracy generally increases. When the number of projection vectors is greater than 10, the classification accuracy increases slowly. By averaging the classification accuracies with different projection vector numbers that are in the range of [1,30], we obtain the overall classification accuracies of PCA and PCA-L1. They are 0.8556 and 0.8552, respectively. The two results are very close, which means that the classification performances of PCA and PCA-L1 are very close. It indicates that incorporating L1-norm into the objective function of PCA, i.e., incorporating robustness into PCA, has little effect on the classification performance, consistent with the results in [14,15].

**Fig 11:**
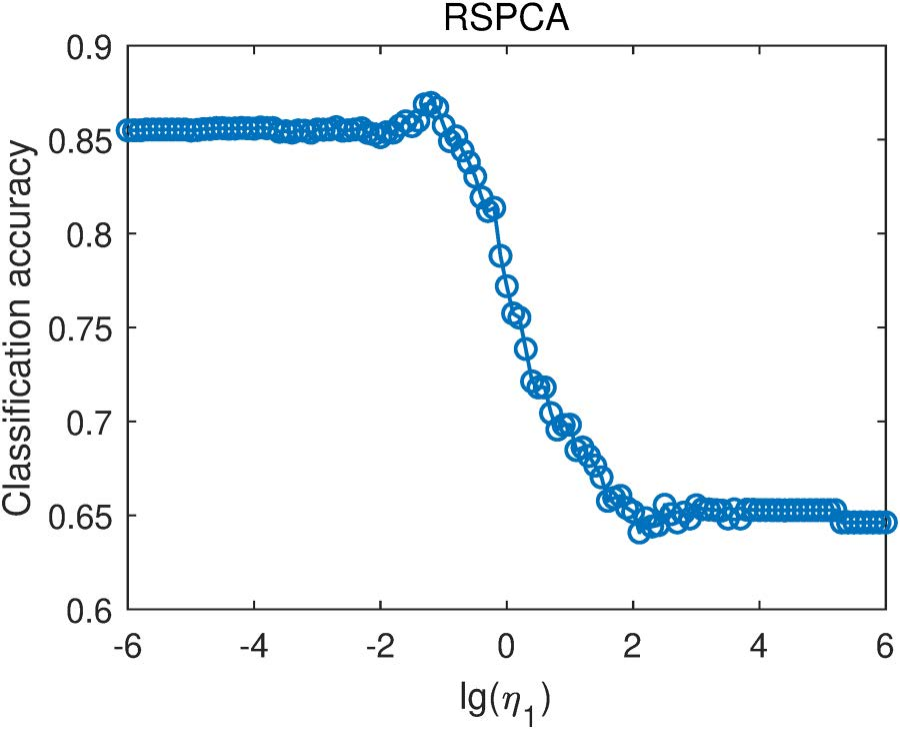
Average classification accuracies of RSPCA with different$\;\mathrm{lg}\left(\eta_{1}\right)$ values.

For RSPCA, the parameters $\eta_{1}$ was selected from $\left\{10^{-6},10^{-5.9},10^{-5.8},\ldots ,10^{6}\right\}$. That is, $\mathrm{lg}\left(\eta_{1}\right)$ was selected in the range of -6–6 with a step of 0.1. For each $\eta_{1}$ value, we averaged the classification accuracies with different projection vector numbers to obtain the overall classification accuracies, as shown in Fig 11. When $\mathrm{lg}\left(\eta_{1}\right)<-1$ or $\mathrm{lg}\left(\eta_{1}\right)>2$, the overall classification accuracies are stable with different values of $\mathrm{lg}\left(\eta_{1}\right)$. When $-1\leq \mathrm{lg}\left(\eta_{1}\right)\leq 2$, the overall classification accuracy generally decreases with increasing $\mathrm{lg}\left(\eta_{1}\right)$ value. The highest classification accuracy is 0.8695, obtained when $\mathrm{lg}\left(\eta_{1}\right)=-1.2$. RSPCA outperforms PCA-L1, indicating that incorporating sparsity improves classification performance, consistent with the results in [14,15].

**Fig 12:**
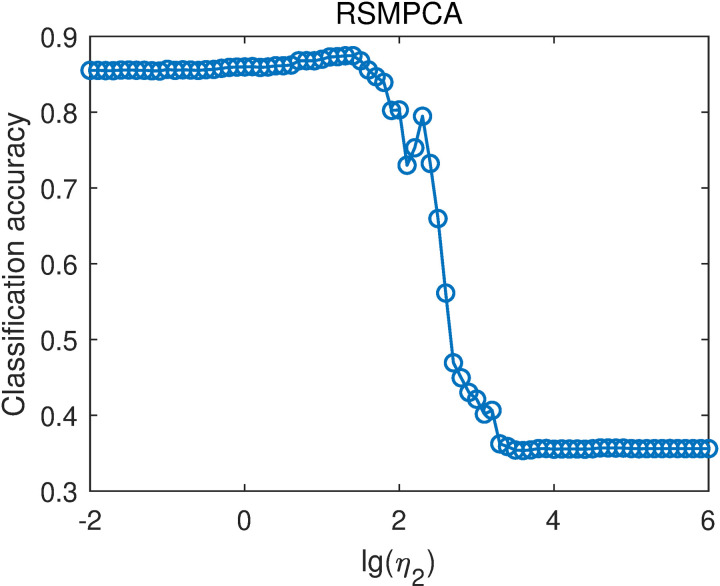
Average classification accuracies of RSMPCA with different $\mathrm{lg}\left(\eta_{2}\right)$ values.

For RSMPCA, the parameter $\eta_{2}$ was selected from $\left\{10^{-2},10^{-1.9},10^{-1.8},\ldots ,10^{6}\right\}$. That is, $\mathrm{lg}\left(\eta_{2}\right)$ was selected in the range of -2–6 with a step of 0.1. The overall classification accuracies of RSMPCA with different $\mathrm{lg}\left(\eta_{2}\right)$ values are shown in Fig 12. When $\mathrm{lg}\left(\eta_{2}\right)<1.4$ or $\mathrm{lg}\left(\eta_{2}\right)>3.3$, the overall classification accuracies are stable with different values of $\mathrm{lg}\left(\eta_{2}\right)$. When $1.4\leq \mathrm{lg}\left(\eta_{2}\right)\leq 3.3$, the overall classification accuracy generally decreases with increasing $\mathrm{lg}\left(\eta_{2}\right)$ value. The highest classification accuracy is 0.8747, obtained when $\mathrm{lg}\left(\eta_{2}\right)=1.4$. RSMPCA outperforms PCA-L1, indicating that incorporating smoothness improves classification performance.

**Fig 13:**
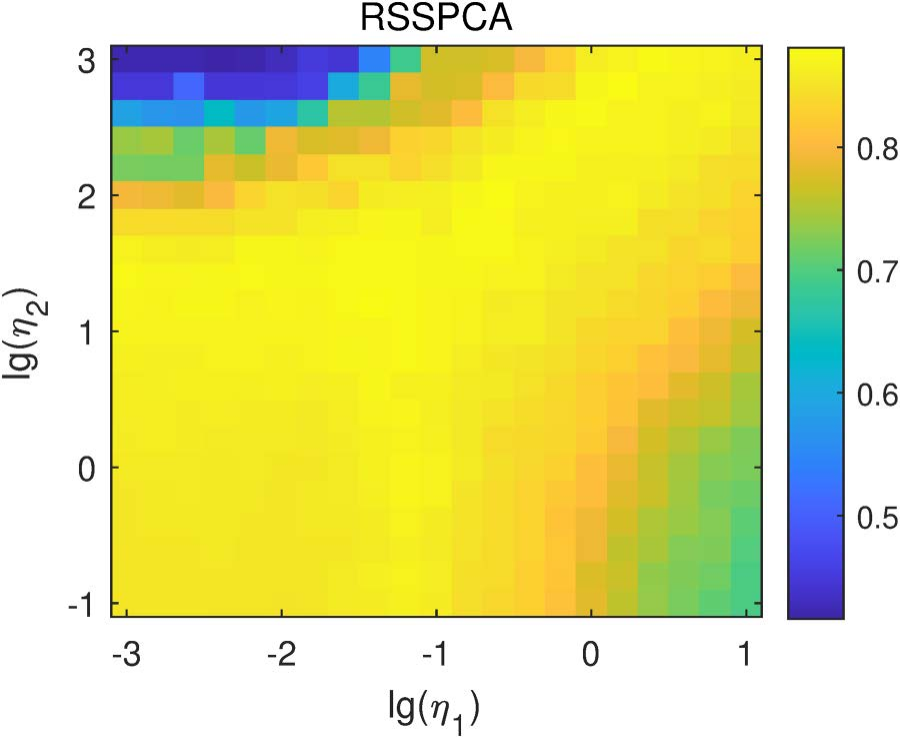
Average classification accuracies of RSSPCA with different $\mathrm{lg}\left(\eta_{1}\right)$ and $\mathrm{lg}\left(\eta_{2}\right)$ values.

For RSSPCA, two parameters were selected according to the classification results of RSPCA and RSMPCA. Specifically, $\mathrm{lg}\left(\eta_{1}\right)$ was selected in the range of -3–1 with a step of 0.2, and $\mathrm{lg}\left(\eta_{1}\right)$ was selected in the range of -1–3 with a step of 0.2. The purpose of setting the step size to 0.2 is to reduce the number of parameter combinations, thereby shortening the total calculation time. The overall classification accuracies of RSSPCA with different parameters are shown in Fig 13. The highest classification accuracy is 0.8810, obtained when $\mathrm{lg}\left(\eta_{1}\right)=-0.6$ and $\mathrm{lg}\left(\eta_{2}\right)=2.0$.

The highest classification accuracies and corresponding optimal parameters of the five algorithms are shown in Table 2. The classification accuracies of PCA and PCA-L1 are very close. It indicates that incorporating L1-norm into the objective function of PCA, i.e., incorporating robustness, has little effect on classification performance. RSPCA incorporates L1-norm constraint into PCA-L1 and achieves a higher classification accuracy than PCA-L1, indicating that incorporating the sparse constraint is beneficial for classification. These results are consistent with the results in [14,15]. RSMPCA incorporates the smooth constraint into PCA-L1 and achieves higher classification accuracy than PCA-L1, indicating that incorporating the smooth constraint is also beneficial for classification. RSSPCA simultaneously incorporates the sparse constraint and the smooth constraint into PCA-L1, and achieves the highest classification accuracy among the five algorithms.

**Table 2:** The highest classification accuracies and optimal parameters of the five algorithms.

Algorithm	Optimal parameters		Classification accuracy
	$\mathrm{lg}\left(\eta_{1}\right)$	$\mathrm{lg}\left(\eta_{2}\right)$	
PCA	/	/	0.8556
PCA-L1	/	/	0.8552
RSPCA	-1.2	/	0.8695
RSMPCA	/	1.4	0.8747
RSSPCA	-0.6	2.0	**0.8810**

In addition, RSSPCA can be generated by incorporating the sparse constraint into RSMPCA. With this adaptation, RSSPCA achieves a higher classification accuracy than RSMPCA. It further demonstrates that incorporating the sparse constraint is beneficial for classification. In another viewpoint, RSSPCA can be generated by incorporating the smooth constraint into RSPCA. With this adaptation, RSSPCA achieves a higher classification accuracy than RSPCA. It further demonstrates that incorporating the smooth constraint is also beneficial for classification.

In summary, incorporating robustness has little effect on classification performance, while incorporating either sparsity or smoothness improves classification performance. Therefore, RSSPCA has advantages over the other four algorithms in face recognition.

### 4.3 Experiments on five additional face databases

To further demonstrate the reconstruction and classification performance of the proposed algorithm, we conducted similar experiments on five additional benchmark face databases, i.e., the AR face database [48], the FEI face database [49], the FERET face database [50], the GT face database [51], and the Yale face database [52]. These datasets are publicly available online and widely distributed for research purposes. The comprehensive details of these face databases, along with their corresponding experimental results, are fully documented in S1 Text within the Supporting Information section.

Table 3 summarizes the lowest reconstruction error and highest classification accuracy of the five algorithms on these face databases. It also includes the corresponding optimal parameters and running time (in seconds) required to compute the projection matrix for each experiment. Additionally, results of the ORL face database are included to provide a comprehensive comparison.

**Table 3:** The reconstruction error and classification accuracy of the five algorithms on the six face databases.

Face database	Algorithm	Reconstruction				Classification			
		$\mathrm{lg}(\eta_{1})$	$\mathrm{lg}(\eta_{2})$	Error	Time(s)	$\mathrm{lg}(\eta_{1})$	$\mathrm{lg}(\eta_{2})$	Accuracy	Time(s)
AR	PCA	/	/	**1145.12**	3.58	/	/	0.5091	3.12
	PCA-L1	/	/	1151.14	11.81	/	/	0.5104	8.98
	RSPCA	-3.0	/	1151.97	57.21	0.1	/	0.6438	482.04
	RSMPCA	/	-1.2	1149.62	62.21	/	-0.1	0.5133	70.95
	RSSPCA	-3.0	-1.0	1150.19	69.18	1.0	0.8	**0.6646**	629.08
FEI	PCA	/	/	513.13	0.45	/	/	0.6245	0.28
	PCA-L1	/	/	510.90	2.13	/	/	0.6270	1.45
	RSPCA	-3.0	/	513.64	7.04	-1.5	/	0.6300	13.83
	RSMPCA	/	-1.0	**510.49**	9.02	/	0.6	0.6482	13.26
	RSSPCA	-3.0	-1.0	510.77	8.85	-1.6	0.8	**0.6541**	17.52
FERET	PCA	/	/	502.88	1.00	/	/	0.2545	0.30
	PCA-L1	/	/	474.41	4.00	/	/	0.2518	2.51
	RSPCA	-3.0	/	474.55	31.98	-0.2	/	0.3142	247.58
	RSMPCA	/	-0.6	**461.52**	48.35	/	1.5	0.3619	32.78
	RSSPCA	-3.0	-0.6	461.86	49.61	-2.2	1.6	**0.3659**	56.06
GT	PCA	/	/	784.46	0.19	/	/	0.6972	0.06
	PCA-L1	/	/	745.06	1.84	/	/	0.6920	1.15
	RSPCA	-3.0	/	746.66	22.90	-1.3	/	0.7025	62.10
	RSMPCA	/	-0.2	**720.18**	27.37	/	1.1	**0.7365**	38.32
	RSSPCA	-2.8	-0.2	720.23	34.54	-3.0	1.2	0.7362	42.85
ORL	PCA	/	/	1272.33	0.06	/	/	0.8556	0.04
	PCA-L1	/	/	1201.79	3.27	/	/	0.8552	2.76
	RSPCA	-3.0	/	1205.41	104.32	-1.2	/	0.8695	428.65
	RSMPCA	/	-0.2	**1152.25**	157.35	/	1.4	0.8747	136.01
	RSSPCA	-3.0	0.0	1153.35	167.41	-0.6	2.0	**0.8810**	513.86
Yale	PCA	/	/	1344.22	0.01	/	/	0.6804	0.01
	PCA-L1	/	/	1231.39	1.95	/	/	0.6822	1.74
	RSPCA	-3.0	/	1237.73	73.22	-2.7	/	0.6838	57.90
	RSMPCA	/	-0.2	1161.28	86.59	/	0.5	**0.6887**	63.40
	RSSPCA	-2.8	-0.2	**1152.87**	118.02	-2.6	0.4	0.6877	95.82

Overall, the running time of PCA and PCA-L1 is much shorter than that of RSPCA, RSMPCA, and RSSPCA. This is because the projection vector w in the latter three algorithms is iteratively updated by Equations (32) and (33), which is a time-consuming process.

In the face reconstruction experiment, the results on the AR and FEI face databases differ from those on the other four face databases. On the AR face database, PCA obtains the lowest reconstruction error among the five competing algorithms. However, on the other five face databases, the lowest reconstruction error is obtained by RSMPCA or RSSPCA. This discrepancy may be attributed to the intrinsic properties of the AR face database. On the FEI face database, the reconstruction errors obtained by PCA-L1, RSMPCA, and RSSPCA are lower than those obtained by PCA and RSPCA. This result is slightly different from those obtained on the other face databases.

On the FERET, GT, and Yale face databases, RSMPCA or RSSPCA obtains the lowest reconstruction errors, while PCA obtains the highest reconstruction errors. Furthermore, the reconstruction errors of PCA-L1 and RSPCA are close, as are those of RSMPCA and RSSPCA. These findings are consistent with those obtained on the ORL face database. In summary, except for the results on the AR and FEI face databases, the results on the other four face databases demonstrate that incorporating robustness and smoothness improves reconstruction performance, while incorporating sparsity has little effect on reconstruction performance.

In the face recognition experiment, except for the results on the AR face database, the lowest classification accuracies are obtained by PCA or PCA-L1, while the highest classification accuracies are obtained by RSMPCA or RSSPCA. On the AR face database, the classification accuracy of RSMPCA is much lower than that of RSSPCA. Again, this may be attributed to the intrinsic property of the AR face database. In general, incorporating sparsity and smoothness improves classification performance, while incorporating robustness has little effect on classification performance.

In summary, the incorporation of robustness and smoothness enhances reconstruction performance, and the incorporation of sparsity and smoothness enhances classification performance. Table 4 summarizes the impact of the three factors, i.e., robustness, sparsity, and smoothness, on reconstruction and classification performance.

**Table 4:** The impact of the three factors on reconstruction and classification performance.

	Reconstruction	Classification
Robustness	√	
Sparsity		√
Smoothness	√	√

## 5. Discussion

This study has three major limitations, as detailed below.

First, identifying the optimal parameters for RSSPCA remains an unresolved issue. This challenge is not unique to RSSPCA but is also present in other parameter-dependent algorithms, such as RSPCA [12,14] and RSMPCA. An exception exists when RSPCA is used for face reconstruction. As indicated in Table 3, RSPCA achieves the lowest reconstruction error with $\text{lg}\left(\eta_{1}\right)\;=\;-3.0$ across all face databases. In this case, RSPCA approximates PCA-L1, which aligns with the findings in [14,15], demonstrating that incorporating sparsity does not affect reconstruction. However, outside of this specific case, determining the optimal parameters for RSPCA, RSMPCA, and RSSPCA is challenging because their results vary across different face databases.

Second, while some general conclusions can be drawn from the experimental results, they do not fully align with our expectations. Notably, on the AR face database, PCA achieves the lowest reconstruction error, and the classification accuracy of RSMPCA is much lower than that of RSSPCA. These outcomes are not observed in the other five databases. This discrepancy suggests that the AR face database has intrinsic properties influencing the results.

Third, the running time of RSPCA, RSMPCA, and RSSPCA is much longer than that of PCA and PCA-L1. This is due to the time-consuming update rule used by the first three algorithms, as specified in Equations (32) and (33). The running time of RSPCA can be reduced by using either of the two update rules in Equations (6)–(11). However, for RSMPCA and RSSPCA, no acceleration strategies have been identified yet due to the incorporation of the smooth constraint.

Potential improvements for the current study are outlined as follows.

First, the smooth constraint in this paper only considers the relationships between spatially adjacent pixels. Extending it to a more general form [41,53] that additionally considers the relations between spatially distant pixels can make full use of the two-dimensional spatial structure information of images.

Second, the robustness and sparsity in this paper are achieved by incorporating L1-norm into the objective function and the constraint function, respectively, in the optimization problem of RSSPCA. They can be further enhanced by replacing L1-norm with an arbitrary norm, i.e., Lp-norm [15,43].

Third, this study only compares five algorithms, i.e., PCA, PCA-L1, RSPCA, RSMPCA, and RSSPCA. However, by incorporating sparsity or smoothness into traditional PCA, we can construct three additional algorithms: Sparse PCA [54], Smooth PCA, and Sparse Smooth PCA. These algorithms can be solved similarly within the MM framework. This paper focuses on improving RSPCA by incorporating the smooth constraint. Therefore, the three non-robust algorithms are not investigated.

As for application of RSSPCA, while this paper focuses exclusively on face reconstruction and recognition, the algorithm is versatile and can be extended to analyze various types of data. For example, RSSPCA can be employed to analyze one-dimensional time series data [55], such as stock prices, heart rate recordings, daily electricity usage, hourly traffic volume, etc. In these cases, the smooth constraint can capture the one-dimensional temporal structure information of the data. For two-dimensional images other than face images, RSSPCA is undoubtedly applicable. Additionally, for high-dimensional data like EEG, MEG, and fMRI [23,25–29], the smooth constraint in RSSPCA can capture both spatial and temporal structure information within these datasets. In conclusion, RSSPCA shows great promise for reconstructing or classifying data that contains spatial or temporal structural information.

## 6. Conclusion

This paper proposes a new algorithm, termed RSSPCA (Robust Sparse Smooth Principal Component Analysis), which enhances RSPCA by incorporating a smooth constraint that captures the two-dimensional spatial structure information of images. An iterative optimization procedure is designed within the Majorization-Minimization (MM) framework to solve the RSSPCA optimization problem.

We evaluate RSSPCA’s performance against four existing algorithms: PCA, PCA-L1, RSPCA, and RSMPCA. Experimental results reveal distinct effects of different constraints: incorporating sparsity has minimal impact on reconstruction performance, while robustness and smoothness contribute to improved reconstruction accuracy. Conversely, robustness shows limited influence in classification tasks, while sparsity and smoothness significantly enhance classification performance.

The proposed RSSPCA algorithm demonstrates clear advantages over existing methods in both face reconstruction and recognition by simultaneously integrating three key properties: robustness, sparsity, and smoothness. Visualization of the generalized eigenfaces clearly illustrates how these constraints influence feature extraction: sparsity enables selective feature identification, while smoothness preserves spatial relationships among facial components. Given these promising results, RSSPCA shows great potential for analyzing data with rich spatial or temporal structural information.

## Supporting information

S1 Text Experiments on five additional face databases. (PDF)
