# Supplementary material for: Robust sparse smooth principal component analysis for face reconstruction and recognition
Source: PLoS One. 2025 May 27;20(5):e0323281. doi: 10.1371/journal.pone.0323281 (PMC12111395; doi:10.1371/journal.pone.0323281)
Supplement: S1 Text — (PDF) [file pone.0323281.s001.pdf]

Supporting Information

Robust Sparse Smooth Principal Component Analysis for Face Reconstruction and Recognition

Jing Wang[1,2*], Xiao Xie[1,2], Li Zhang[3], Jian Li[1,2], Hao Cai[1,2], Yan Feng[1,2]

[1] School of Computer and Information Technology, Xinyang Normal University, Xinyang, China.

[2] Henan Key Laboratory of Analysis and Applications of Education Big Data, Xinyang Normal University, Xinyang, China.

[3] School of Early-Childhood Education, Nanjing Xiaozhuang University, Nanjing, Jiangsu, China

***Corresponding author**

E-mail: wangjing@xynu.edu.cn (JW)

Experiments on five additional face databases

To further demonstrate the reconstruction and classification capabilities of the proposed algorithm, we conducted analogous experiments using five additional benchmark face databases. These databases include the AR face database [1], the FEI face database [2], the FERET face database [3], the GT face database [4], and the Yale face database [5]. Four typical PCA-based algorithms, i.e., PCA [6, 7], PCA-L1 [8], RSPCA [9], and RSMPCA, were compared with RSSPCA in the experiment.

In the face reconstruction experiments, we randomly selected 20% of the images from each dataset and applied rectangular salt-and-pepper noise for occlusion. The size of the noise was not smaller than 10 by 10. The position of the noise was located randomly. Then we trained the projection matrix for each algorithm on the polluted face database and calculated the average reconstruction error by Equation (34).

In the face recognition experiments, we randomly selected about 2/3 of the images from each face database for training and used the remaining images for testing. The training images were normalized by z-score so that each feature was centered to have a mean of zero and scaled

to have a standard deviation of one. The testing images were then normalized by applying the same parameters. After that, we applied the five competing algorithms to extract the first 30 projection vectors and applied these projection vectors to reduce the dimension of the normalized data. Finally, Nearest Neighbor (NN) classifier [10] was applied to perform classification. The above procedure was repeated three times, and the average classification accuracies were calculated to evaluate the classification performance of the five algorithms.

Table S1 shows the main information of the face databases used in the experiments. The information of the ORL face database [11, 12] is also included in this table for comparison.

Table S1. The main information of the face databases used in the experiments.

Database	AR	FEI	FERET	GT	ORL	Yale
Height	50	24	40	43	56	50
Width	40	32	40	32	46	50
No. of images	3120	2800	1400	750	400	165
No. of subjects	120	200	200	50	40	15
No. of images per subject	26	14	7	15	10	11
No. of occluded images	624	560	280	150	80	33
No. of clean images	2496	2240	1120	600	320	132
No. of training images per subject	18	10	5	10	7	8
No. of testing images per subject	8	4	2	5	3	3

Detailed information on the five additional face databases, along with corresponding face reconstruction and recognition results, are provided below to support the conclusions presented in the main text.

AR face database

The AR face database [1] contains 3120 face images from 120 subjects, 26 images per subject. The images were taken in two different sessions separated by two weeks, and 13 images were taken in each session for each subject. Additionally, the images were captured with different facial expressions and illuminations, and some of them are occluded with black sunglasses or towels. The image size is 50 by 40.

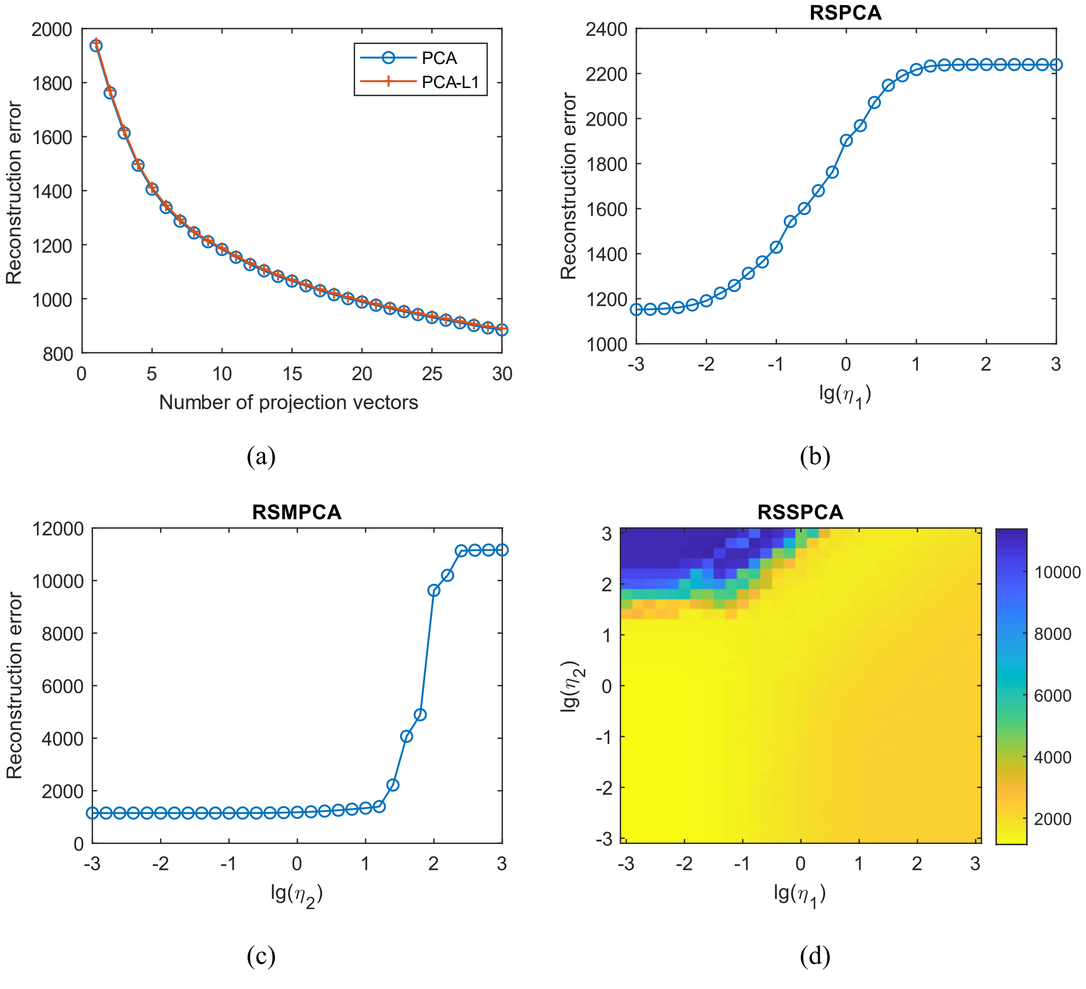

Fig S1. Reconstruction errors on the AR face database.

The reconstruction errors of the five algorithms on the AR face database are shown in Fig S1. Fig S1(a) shows the reconstruction errors of PCA and PCA-L1 with different numbers of projection vectors. The two curves closely overlap. The average reconstruction errors of PCA and PCA-L1 are 1145.12 and 1151.14, respectively. Fig S1(b) shows the reconstruction errors

of RSPCA with different $\lg(\eta_1)$ values. The lowest reconstruction error is 1151.97, obtained when $\lg(\eta_1) = -3.0$. Fig S1(c) shows the reconstruction errors of RSMPCA with different $\lg(\eta_2)$ values. The lowest reconstruction error is 1149.62, obtained when $\lg(\eta_2) = -1.2$. Fig S1(d) shows the reconstruction errors of RSSPCA with different $\lg(\eta_1)$ and $\lg(\eta_2)$ values. The lowest reconstruction error is 1150.19, obtained when $\lg(\eta_1) = -3.0$ and $\lg(\eta_2) = -1.0$.

The lowest reconstruction errors and corresponding optimal parameters of the five algorithms are shown in Table S2. It can be inferred that incorporating robustness and smoothness improves reconstruction performance, while incorporating sparsity has limited effect on reconstruction performance.

Table S2. The lowest reconstruction errors and optimal parameters on the AR face database.

**Algorithm**	**Optimal parameters**		**Reconstruction error**
	$\lg(\eta_1)$	$\lg(\eta_2)$	
PCA	/	/	1145.12
PCA-L1	/	/	1151.14
RSPCA	-3.0	/	1151.97
RSMPCA	/	-1.2	1149.62
RSSPCA	-3.0	-1.0	1150.19

The classification accuracies of the five algorithms on the AR face database are shown in Fig S2. Fig S2(a) shows the classification accuracies of PCA and PCA-L1 with different numbers of projection vectors. The two curves closely overlap. The average classification accuracies of PCA and PCA-L1 are 0.5091 and 0.5104, respectively. Fig S2(b) shows the classification accuracies of RSPCA with different $\lg(\eta_1)$ values. The highest classification accuracy is 0.6438, obtained when $\lg(\eta_1) = 0.1$. Fig S2(c) shows the classification accuracies of RSMPCA with different $\lg(\eta_2)$ values. The highest classification accuracy is 0.5133, obtained when $\lg(\eta_2) = -0.1$. Fig S2(d) shows the classification accuracies of RSSPCA with different $\lg(\eta_1)$ and $\lg(\eta_2)$ values. The highest classification accuracy is 0.6646, obtained when $\lg(\eta_1) = 1.0$ and $\lg(\eta_2) = 0.8$.

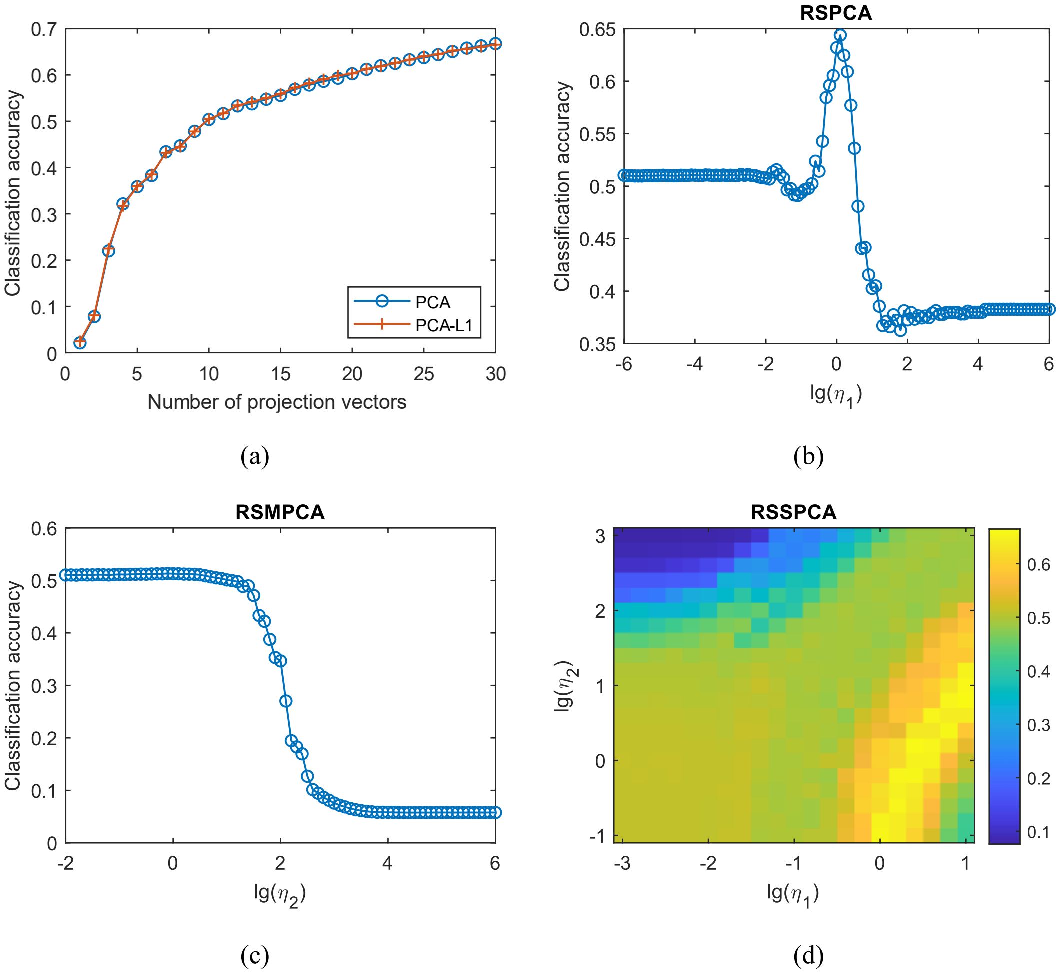

Fig S2. Classification accuracies on the AR face database.

The highest classification accuracies and corresponding optimal parameters of the five algorithms are shown in Table S3. It can be inferred that incorporating robustness has limited effect on classification performance, while incorporating sparsity and smoothness improves classification performance.

Table S3. The highest classification accuracies and optimal parameters on the AR face database.

Algorithm	Optimal parameters		Classification accuracy
	$\lg(\eta_1)$	$\lg(\eta_2)$	
PCA	/	/	0.5091
PCA-L1	/	/	0.5104
RSPCA	0.1	/	0.6438
RSMPCA	/	-0.1	0.5133
RSSPCA	1.0	0.8	0.6646

FEI face database

The FEI face database [2] contains 2800 face images from 120 subjects, 14 images per subject. The images were captured with different view angles and illuminations. The image size is 48 by 46. We further resize the images to 24 by 32 to reduce computational time.

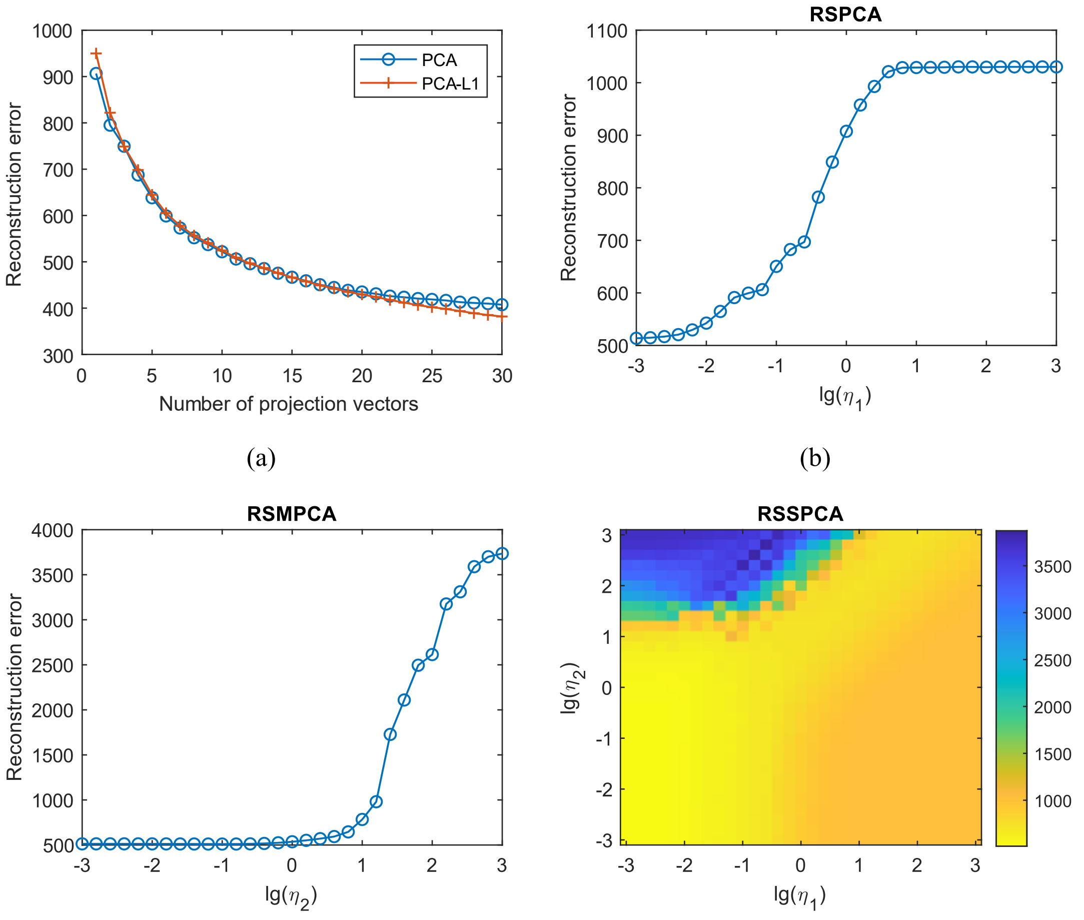

(c) (d)

Fig S3. Reconstruction errors on the FEI face database.

The reconstruction errors of the five algorithms on the FEI face database are shown in Fig S3. Fig S3(a) shows the reconstruction errors of PCA and PCA-L1 with different numbers of projection vectors. The average reconstruction errors of PCA and PCA-L1 are 513.13 and 510.90, respectively. Fig S3(b) shows the reconstruction errors of RSPCA with different $\lg(\eta_1)$ values. The lowest reconstruction error is 513.64, obtained when $\lg(\eta_1) = -3.0$. Fig S3(c) shows the reconstruction errors of RSMPCA with different $\lg(\eta_2)$ values. The lowest

reconstruction error is 510.49, obtained when $\lg(\eta_2) = -1.0$. Fig S3(d) shows the reconstruction errors of RSSPCA with different $\lg(\eta_1)$ and $\lg(\eta_2)$ values. The lowest reconstruction error is 510.77, obtained when $\lg(\eta_1) = -3.0$ and $\lg(\eta_2) = -1.0$.

The lowest reconstruction errors and corresponding optimal parameters of the five algorithms are shown in Table S4. It can be inferred that incorporating robustness and smoothness improves reconstruction performance, while incorporating sparsity has limited effect on reconstruction performance.

Table S4. The lowest reconstruction errors and optimal parameters on the FEI face database.

Algorithm	Optimal parameters		Reconstruction error
	$\lg(\eta_1)$	$\lg(\eta_2)$	
PCA	/	/	513.13
PCA-L1	/	/	510.90
RSPCA	-3.0	/	513.64
RSMPCA	/	-1.0	510.49
RSSPCA	-3.0	-1.0	510.77

The classification accuracies of the five algorithms on the FEI face database are shown in Fig S4. Fig S4(a) shows the classification accuracies of PCA and PCA-L1 with different numbers of projection vectors. The two curves closely overlap. The average classification accuracies of PCA and PCA-L1 are 0.6245 and 0.6270, respectively. Fig S4(b) shows the classification accuracies of RSPCA with different $\lg(\eta_1)$ values. The highest classification accuracy is 0.6300, obtained when $\lg(\eta_1) = -1.5$. Fig S4(c) shows the classification accuracies of RSMPCA with different $\lg(\eta_2)$ values. The highest classification accuracy is 0.6482, obtained when $\lg(\eta_2) = 0.6$. Fig S4(d) shows the classification accuracies of RSSPCA with different $\lg(\eta_1)$ and $\lg(\eta_2)$ values. The highest classification accuracy is 0.6541, obtained when $\lg(\eta_1) = -1.6$ and $\lg(\eta_2) = 0.8$.

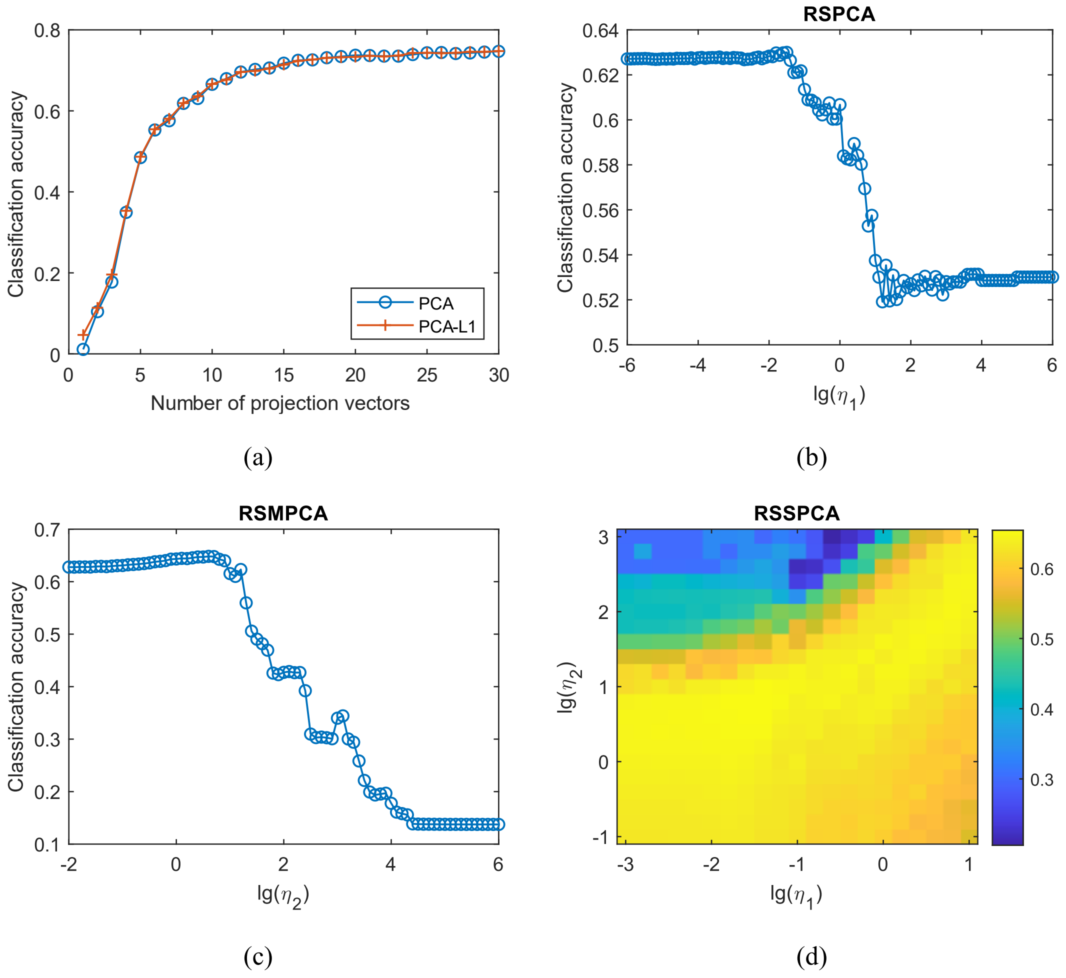

Fig S4. Classification accuracies on the FEI face database.

The highest classification accuracies and corresponding optimal parameters of the five algorithms are shown in Table S5. It can be inferred that incorporating robustness has limited effect on classification performance, while incorporating sparsity and smoothness improves classification performance.

Table S5. The highest classification accuracies and optimal parameters on the FEI face database.

Algorithm	Optimal parameters		Classification accuracy
	lg(η_1)	lg(η_2)	
PCA	/	/	0.6245
PCA-L1	/	/	0.6270
RSPCA	-1.5	/	0.6300
RSMPCA	/	0.6	0.6482
RSSPCA	-1.6	0.8	0.6541

FERET face database

The FERET face database [3] contains 1400 face images from 200 subjects, 7 images per subject. The images were captured with different facial expressions, view angles, and illuminations. The image size is 80 by 80. We further resize the images to 40 by 40 to reduce computational time.

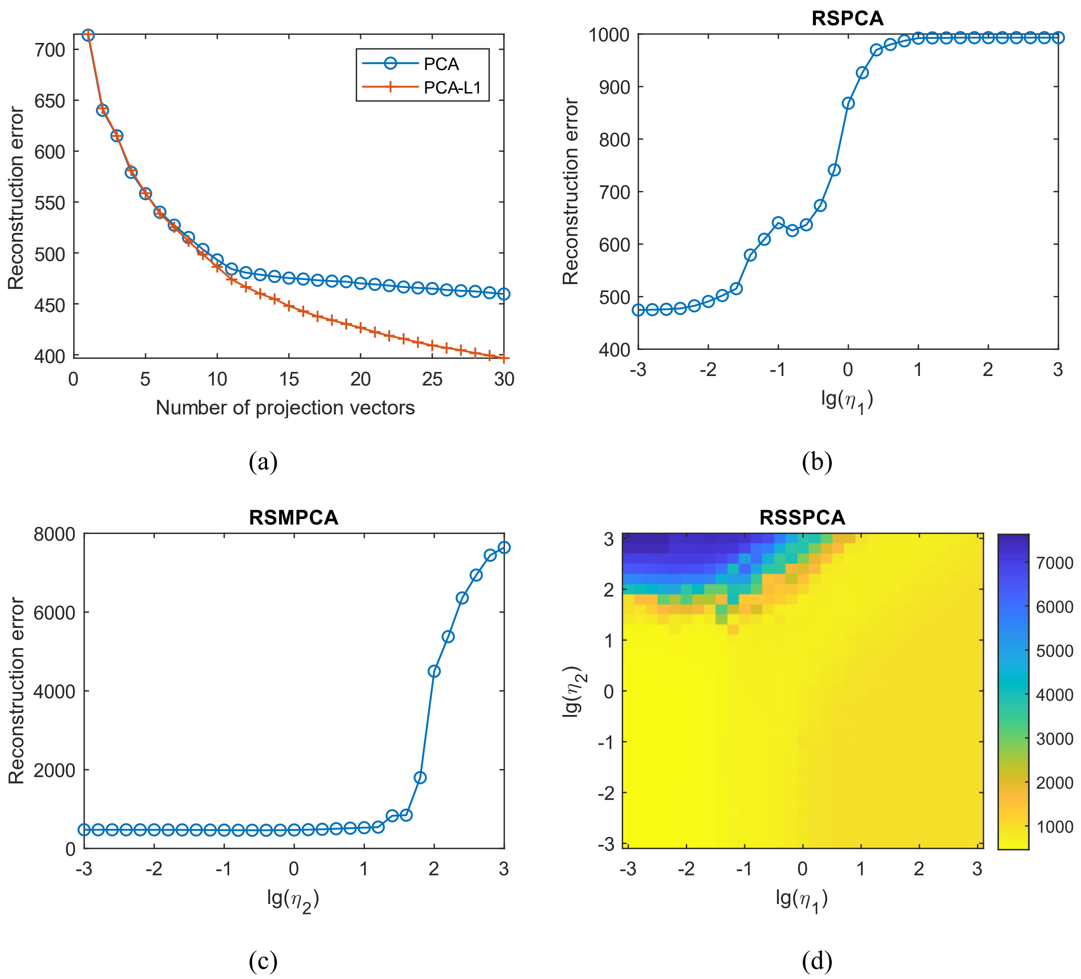

Fig S5. Reconstruction errors on the FERET face database.

The reconstruction errors of the five algorithms on the FERET face database are shown in Fig S5. Fig S5(a) shows the reconstruction errors of PCA and PCA-L1 with different numbers of projection vectors. The average reconstruction errors of PCA and PCA-L1 are 502.88 and 474.41, respectively. Fig S5(b) shows the reconstruction errors of RSPCA with different $\lg(\eta_1)$ values. The lowest reconstruction error is 474.55, obtained when $\lg(\eta_1) = -3.0$. Fig

S5(c) shows the reconstruction errors of RSMPCA with different $\lg(\eta_2)$ values. The lowest reconstruction error is 461.52, obtained when $\lg(\eta_2) = -0.6$. Fig S5(d) shows the reconstruction errors of RSSPCA with different $\lg(\eta_1)$ and $\lg(\eta_2)$ values. The lowest reconstruction error is 461.86, obtained when $\lg(\eta_1) = -3.0$ and $\lg(\eta_2) = -0.6$.

The lowest reconstruction errors and corresponding optimal parameters of the five algorithms are shown in Table S6. It can be inferred that incorporating robustness and smoothness improves reconstruction performance, while incorporating sparsity has limited effect on reconstruction performance.

Table S6. The lowest reconstruction errors and optimal parameters on the FERET face database.

Algorithm	Optimal parameters		Reconstruction error
	$\lg(\eta_1)$	$\lg(\eta_2)$	
PCA	/	/	502.88
PCA-L1	/	/	474.41
RSPCA	-3.0	/	474.55
RSMPCA	/	-0.6	461.52
RSSPCA	-3.0	-0.6	461.86

The classification accuracies of the five algorithms on the FERET face database are shown in Fig S6. Fig S6(a) shows the classification accuracies of PCA and PCA-L1 with different numbers of projection vectors. The two curves closely overlap. The average classification accuracies of PCA and PCA-L1 are 0.2545 and 0.2518, respectively. Fig S6(b) shows the classification accuracies of RSPCA with different $\lg(\eta_1)$ values. The highest classification accuracy is 0.3142, obtained when $\lg(\eta_1) = -0.2$. Fig S6(c) shows the classification accuracies of RSMPCA with different $\lg(\eta_2)$ values. The highest classification accuracy is 0.3619, obtained when $\lg(\eta_2) = 1.5$. Fig S6(d) shows the classification accuracies of RSSPCA with different $\lg(\eta_1)$ and $\lg(\eta_2)$ values. The highest classification accuracy is 0.3659, obtained when $\lg(\eta_1) = -2.2$ and $\lg(\eta_2) = 1.6$.

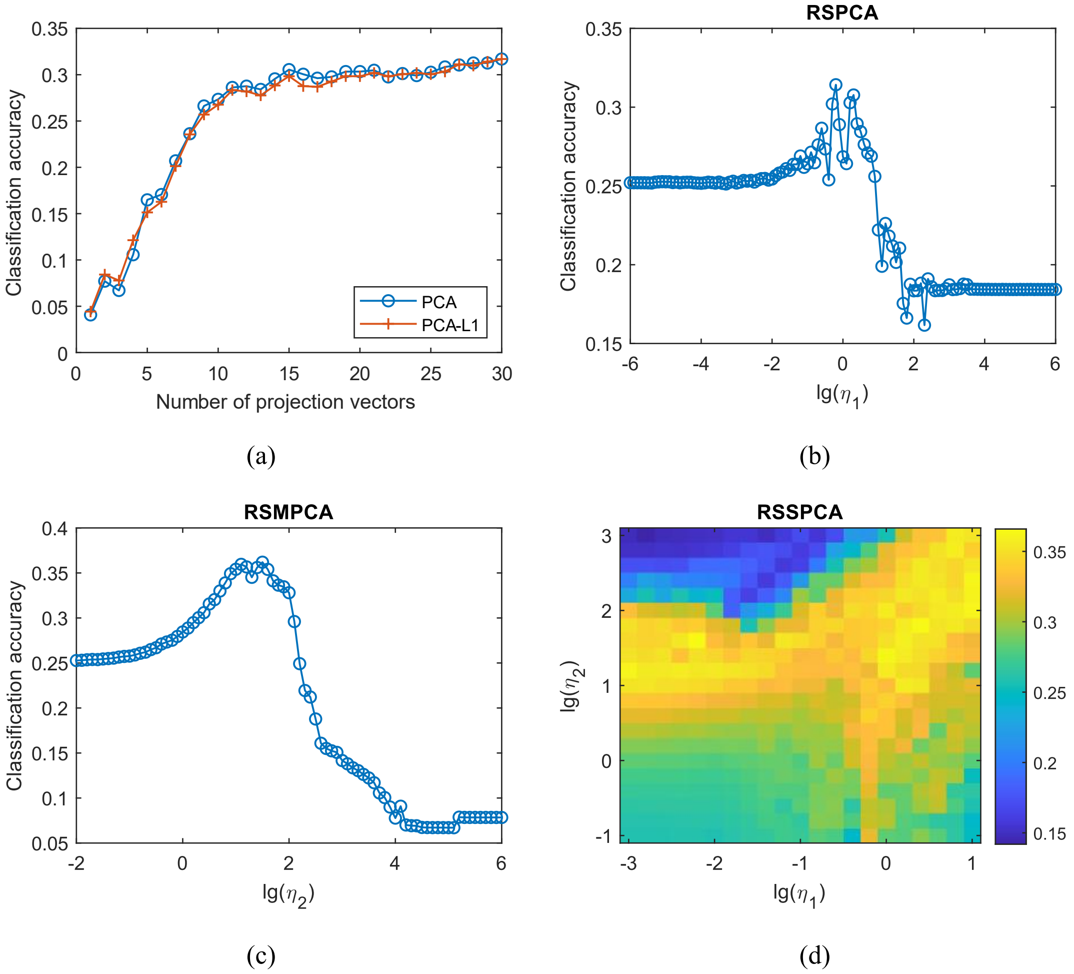

Fig S6. Classification accuracies on the FERET face database.

The highest classification accuracies and corresponding optimal parameters of the five algorithms are shown in Table S7. It can be inferred that incorporating robustness has limited effect on classification performance, while incorporating sparsity and smoothness improves classification performance.

Table S7. The highest classification accuracies and optimal parameters on the FERET face database.

Algorithm	Optimal parameters lg(η_1)	Optimal parameters lg(η_2)	Classification accuracy
PCA	/	/	0.2545
PCA-L1	/	/	0.2518
RSPCA	-0.2	/	0.3142
RSMPCA	/	1.5	0.3619
RSSPCA	-2.2	1.6	0.3659

GT face database

The GT face database [4] contains 750 face images from 50 subjects, 15 images per subject. The images were captured with different facial expressions, view angles, and illuminations. The image size is resized to 43 by 32.

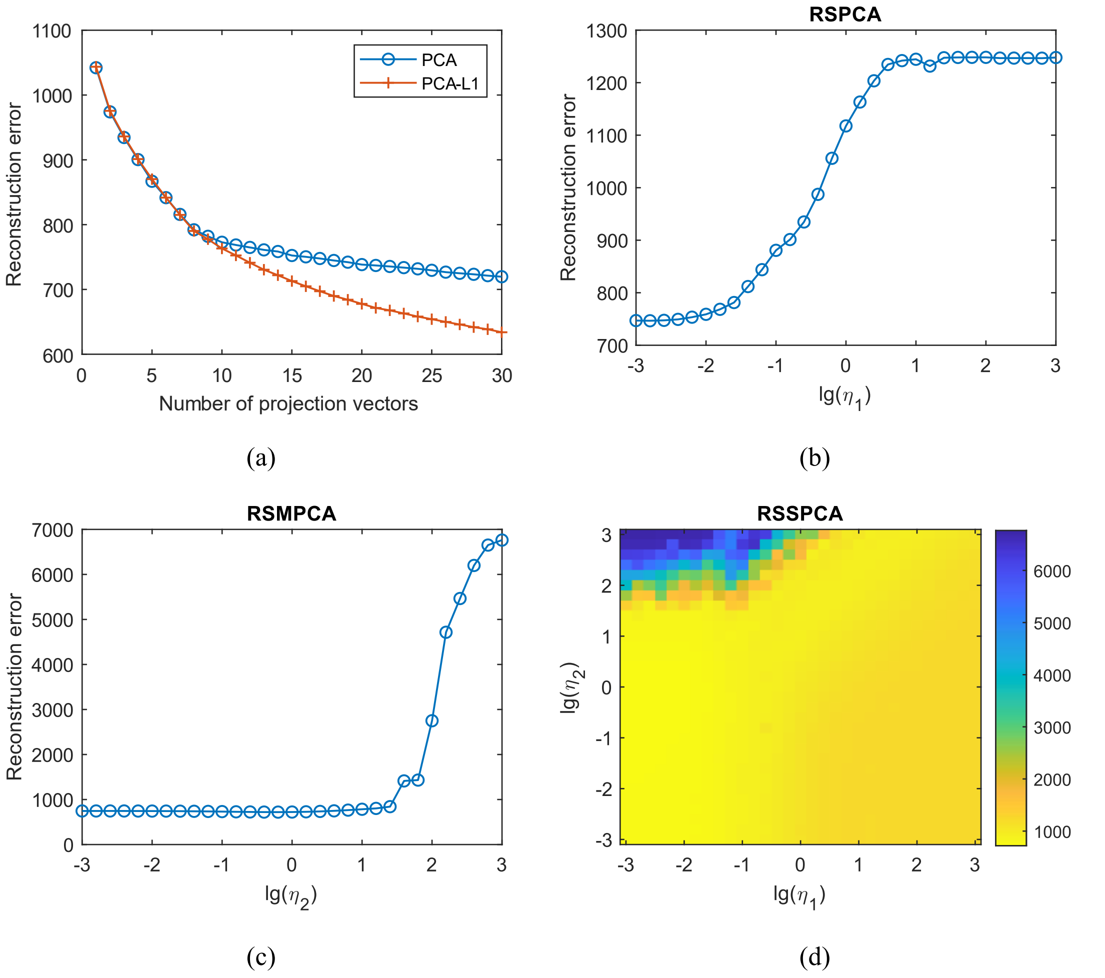

Fig S7. Reconstruction errors on the GT face database.

The reconstruction errors of the five algorithms on the GT face database are shown in Fig S7. Fig S7(a) shows the reconstruction errors of PCA and PCA-L1 with different numbers of projection vectors. The average reconstruction errors of PCA and PCA-L1 are 784.46 and 745.06, respectively. Fig S7(b) shows the reconstruction errors of RSPCA with different $\lg(\eta_1)$ values. The lowest reconstruction error is 746.66, obtained when $\lg(\eta_1) = -3.0$. Fig S7(c) shows the reconstruction errors of RSMPCA with different $\lg(\eta_2)$ values. The lowest reconstruction error is 720.18, obtained when $\lg(\eta_2) = -0.2$. Fig S7(d) shows the

reconstruction errors of RSSPCA with different $\lg(\eta_1)$ and $\lg(\eta_2)$ values. The lowest reconstruction error is 720.23, obtained when $\lg(\eta_1) = -2.8$ and $\lg(\eta_2) = -0.2$.

The lowest reconstruction errors and corresponding optimal parameters of the five algorithms are shown in Table S8. It can be inferred that incorporating robustness and smoothness improves reconstruction performance, while incorporating sparsity has limited effect on reconstruction performance.

Table S8. The lowest reconstruction errors and optimal parameters on the GT face database.

**Algorithm**	**Optimal parameters**		**Reconstruction error**
	$\lg(\eta_1)$	$\lg(\eta_2)$	
PCA	/	/	784.46
PCA-L1	/	/	745.06
RSPCA	-3.0	/	746.66
RSMPCA	/	-0.2	720.18
RSSPCA	-2.8	-0.2	720.23

The classification accuracies of the five algorithms on the GT face database are shown in Fig S8. Fig S8(a) shows the classification accuracies of PCA and PCA-L1 with different numbers of projection vectors. The two curves closely overlap. The average classification accuracies of PCA and PCA-L1 are 0.6972 and 0.6920, respectively. Fig S8(b) shows the classification accuracies of RSPCA with different $\lg(\eta_1)$ values. The highest classification accuracy is 0.7025, obtained when $\lg(\eta_1) = -1.3$. Fig S8(c) shows the classification accuracies of RSMPCA with different $\lg(\eta_2)$ values. The highest classification accuracy is 0.7365, obtained when $\lg(\eta_2) = 1.1$. Fig S8(d) shows the classification accuracies of RSSPCA with different $\lg(\eta_1)$ and $\lg(\eta_2)$ values. The highest classification accuracy is 0.7362, obtained when $\lg(\eta_1) = -3.0$ and $\lg(\eta_2) = 1.2$.

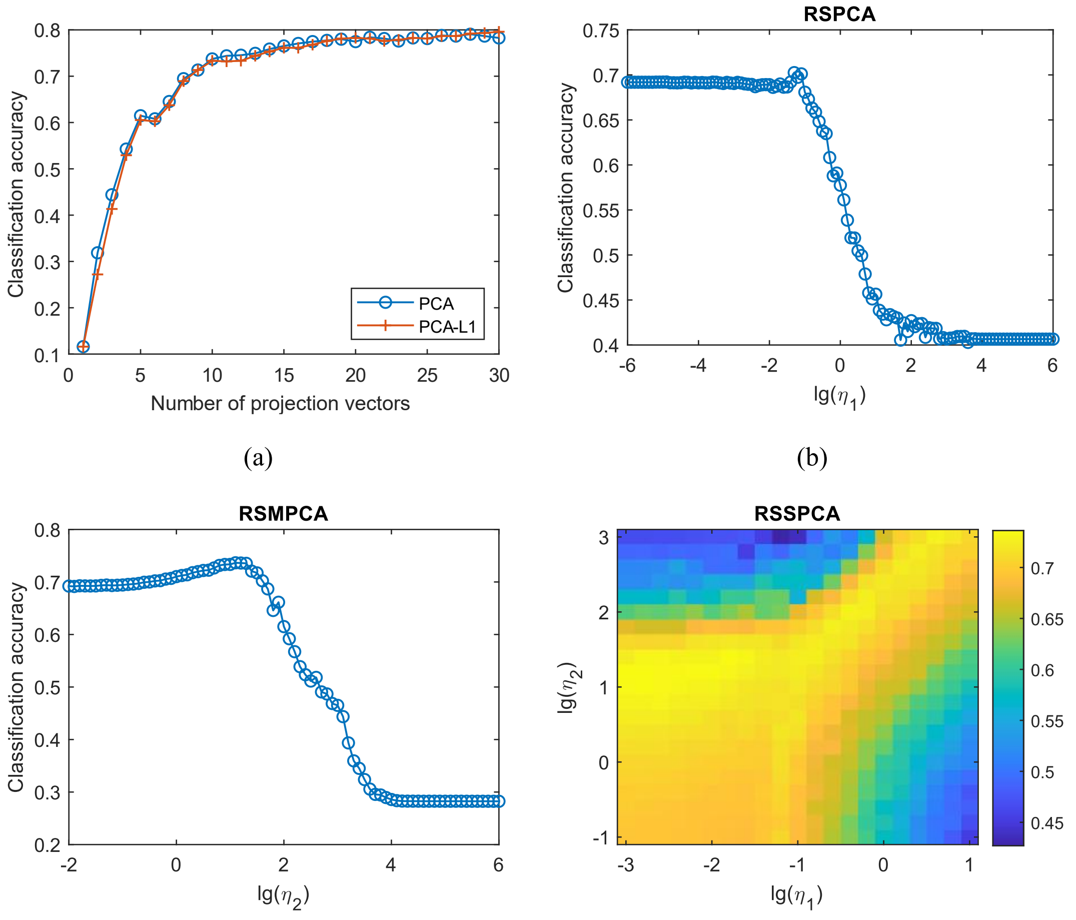

(a) (b)

(c) (d)

Fig S8. Classification accuracies on the GT face database.

The highest classification accuracies and corresponding optimal parameters of the five algorithms are shown in Table S9. It can be inferred that incorporating robustness has limited effect on classification performance, while incorporating sparsity and smoothness improves classification performance.

Table S9. The highest classification accuracies and optimal parameters on the GT face database.

**Algorithm**	**Optimal parameters** lg(η_1)	lg(η_2)	**Classification accuracy**
PCA	/	/	0.6972
PCA-L1	/	/	0.6920
RSPCA	-1.3	/	0.7025
RSMPCA	/	1.1	0.7365
RSSPCA	-3.0	1.2	0.7362

Yale face database

The Yale face database [5] contains 165 face images from 15 subjects, 11 images per subject. The images were captured with different facial expressions, view angles, and illuminations. The image size is 100 by 100. We further resize the images to 50 by 50 to reduce computational time.

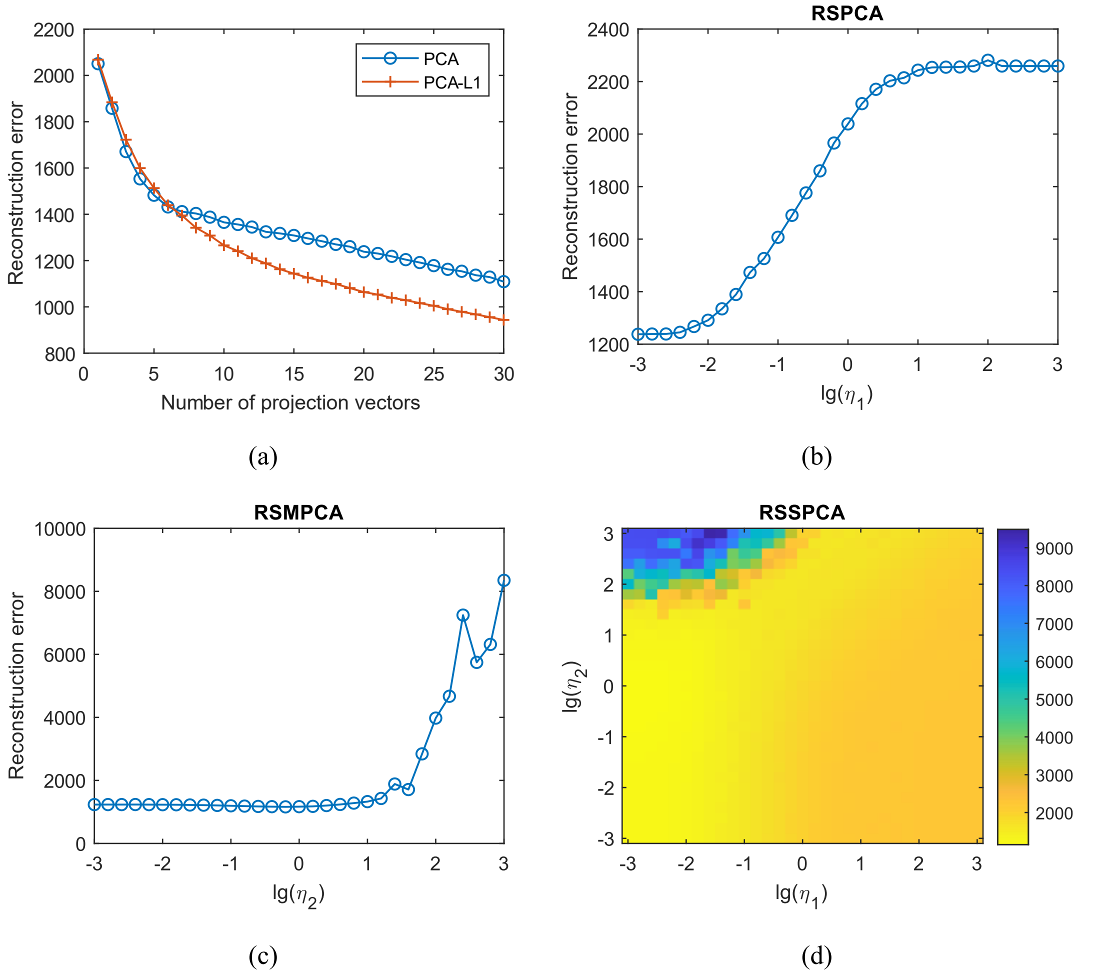

Fig S9. Reconstruction errors on the Yale face database.

The reconstruction errors of the five algorithms on the Yale face database are shown in Fig S9. Fig S9(a) shows the reconstruction errors of PCA and PCA-L1 with different numbers of projection vectors. The average reconstruction errors of PCA and PCA-L1 are 1344.22 and 1231.39, respectively. Fig S9(b) shows the reconstruction errors of RSPCA with different $\lg(\eta_1)$ values. The lowest reconstruction error is 1237.73, obtained when $\lg(\eta_1) = -3.0$. Fig

S9(c) shows the reconstruction errors of RSMPCA with different $\lg(\eta_2)$ values. The lowest reconstruction error is 1161.28, obtained when $\lg(\eta_2) = -0.2$. Fig S9(d) shows the reconstruction errors of RSSPCA with different $\lg(\eta_1)$ and $\lg(\eta_2)$ values. The lowest reconstruction error is 1152.87, obtained when $\lg(\eta_1) = -2.8$ and $\lg(\eta_2) = -0.2$.

The lowest reconstruction errors and corresponding optimal parameters of the five algorithms are shown in Table S10. It can be inferred that incorporating robustness and smoothness improves reconstruction performance, while incorporating sparsity has limited effect on reconstruction performance.

Table S10. The lowest reconstruction errors and optimal parameters on the Yale face database.

Algorithm	Optimal parameters		Reconstruction error
	$\lg(\eta_1)$	$\lg(\eta_2)$	
PCA	/	/	1344.22
PCA-L1	/	/	1231.39
RSPCA	-3.0	/	1237.73
RSMPCA	/	-0.2	1161.28
RSSPCA	-2.8	-0.2	1152.87

The classification accuracies of the five algorithms on the Yale face database are shown in Fig S10. Fig S10(a) shows the classification accuracies of PCA and PCA-L1 with different numbers of projection vectors. The two curves closely overlap. The average classification accuracies of PCA and PCA-L1 are 0.6804 and 0.6822, respectively. Fig S10(b) shows the classification accuracies of RSPCA with different $\lg(\eta_1)$ values. The highest classification accuracy is 0.6838, obtained when $\lg(\eta_1) = -2.7$. Fig S10(c) shows the classification accuracies of RSMPCA with different $\lg(\eta_2)$ values. The highest classification accuracy is 0.6887, obtained when $\lg(\eta_2) = 0.5$. Fig S10(d) shows the classification accuracies of RSSPCA with different $\lg(\eta_1)$ and $\lg(\eta_2)$ values. The highest classification accuracy is 0.6877, obtained when $\lg(\eta_1) = -2.6$ and $\lg(\eta_2) = 0.4$.

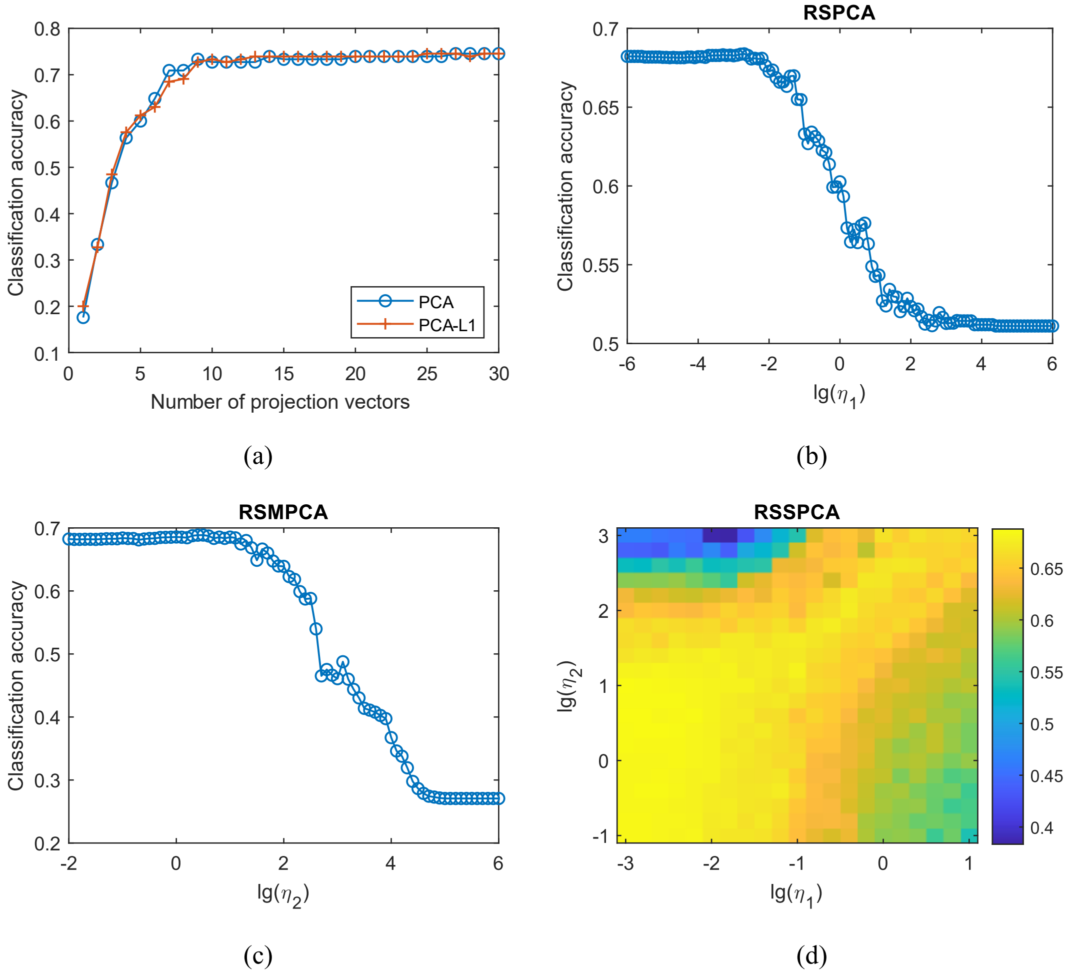

Fig S10. Classification accuracies on the Yale face database.

The highest classification accuracies and corresponding optimal parameters of the five algorithms are shown in Table S11. It can be inferred that incorporating robustness has limited effect on classification performance, while incorporating sparsity and smoothness improves classification performance.

Table S11. The highest classification accuracies and optimal parameters on the Yale face database.

Algorithm	Optimal parameters		Classification accuracy
	$\lg(\eta_1)$	$\lg(\eta_2)$	
PCA	/	/	0.6804
PCA-L1	/	/	0.6822
RSPCA	-2.7	/	0.6838
RSMPCA	/	0.5	0.6887
RSSPCA	-2.6	0.4	0.6877

References

1. Martinez A, Benavente R. The AR face database. CVC Technical Report. 1998;24.
2. Thomaz CE, Giraldi GA. A new ranking method for principal components analysis and its application to face image analysis. Image and Vision Computing. 2010;28(6):902-13.
3. Phillips PJ, Martin A, Wilson CL, Przybocki M. An introduction evaluating biometric systems. Computer. 2000;33(2):56-63.
4. Nefian AV, Hayes MH. Hidden Markov models for face recognition. IEEE International Conference on Acoustics, Speech and Signal Processing; 1998.
5. Georghiades AS, Belhumeur PN, Kriegman DJ. From few to many: Illumination cone models for face recognition under variable lighting and pose. IEEE Transactions on Pattern Analysis and Machine Intelligence. 2001;23(6):643-60.
6. Bharadiya JP. A Tutorial on Principal Component Analysis for Dimensionality Reduction in Machine Learning. International Journal of Innovative Science and Research Technology. 2023;8(5):2028-32.
7. Jolliffe I. A 50-year personal journey through time with principal component analysis. Journal of Multivariate Analysis. 2022;188:104820.
8. Kwak N. Principal Component Analysis Based on L1-Norm Maximization. IEEE Transactions on Pattern Analysis and Machine Intelligence. 2008;30(9):1672-80.
9. Meng D, Zhao Q, Xu Z. Improve Robustness of Sparse PCA by L1-norm Maximization. Pattern Recognition. 2012;45(1):487-97.
10. Cunningham P, Delany SJ. K-nearest neighbour classifiers-a tutorial. ACM computing surveys (CSUR). 2021;54(6):1-25.
11. Samaria FS, Harter AC. Parameterisation of a stochastic model for human face identification. IEEE workshop on applications of computer vision; 1994.
12. Samaria FS. Face recognition using hidden Markov models: University of Cambridge Cambridge, UK; 1994.
